# Computational cognitive modelling of action awareness: prior and retrospective

**DOI:** 10.1007/s40708-015-0013-3

**Published:** 2015-03-20

**Authors:** Dilhan J. Thilakarathne, Jan Treur

**Affiliations:** Agent Systems Research Group, Department of Computer Science, VU University Amsterdam, De Boelelaan 1081, 1081 HV Amsterdam, The Netherlands

**Keywords:** Awareness, Prior, Retrospective, Cognitive modelling

## Abstract

This paper presents a computational cognitive model for action awareness focusing on action preparation and performance by considering its cognitive effects and affects from both prior and retrospective form relative to the action execution. How action selection and execution contribute to the awareness or vice versa is a research question, and from the findings of brain imaging and recording techniques more information has become available on this. Some evidence leads to a hypothesis that awareness of action selection is not directly causing the action execution (or behaviour) but comes afterwards as an effect of unconscious processes of action preparation. In contrast, another hypothesis claims that both predictive and inferential processes related to the action preparation and execution may contribute to the conscious awareness of the action, and furthermore, this awareness of an action is a dynamic combination of both prior awareness (through predictive motor control processes) and retrospective awareness (through inferential sense-making processes) relative to the action execution. The proposed model integrates the findings of both conscious and unconscious explanations for both action awareness and ownership and acts as a generic computational cognitive model to explain agent behaviour through the interplay between conscious and unconscious processes. Validation of the proposed model is achieved through simulations on suitable scenarios which are covered with actions that are prepared without being conscious at any point in time, and also with the actions that agent develops prior awareness and/or retrospective awareness. Having selected an interrelated set of scenarios, a systematic approach is used to find a suitable but generic parameter value set which is used throughout all the simulations that highlights the strength of the design of this cognitive model.

## Introduction

Humans intuitively feel that their behaviour is an effect of their conscious decisions for certain actions aiming for desired outcomes [1]. However, what exactly is consciousness is a well-known question among many scientists in many disciplines (see [2, 3]); for example, is it just a process in the brain, and if so, how is it composed? With the developments in brain imaging and recording techniques, more and more detailed information on various brain processes becomes available, including the conscious awareness of actions. One of the leading hypotheses for action awareness is that humans may prepare for and perform actions without being conscious of these preparation and execution processes. More specifically, the feeling of intention for an action is not causing the behaviour but comes after the action preparation and just before the action execution time [4–8]. It has been found that for certain types of actions, the decision to perform it is already made at least hundreds of milliseconds (and even up to 10 s) before any awareness state occurs [4, 5, 8]. The brain predicts the outcome of a decision even before the decision reaches awareness and humans’ (illusionary) consciousness seems like an after-effect of a set of unconscious cognitive processes leading to the action [1, 4–8].

The human brain is a complex, intricate, adaptive, dynamic system; it is difficult to unravel it and comprehend its mechanisms (cf. [9–14]). Therefore, given the complexity and contradictions observed in different experiments, alternative hypotheses are also proposed on action awareness (cf. [15–19]). In particular, it is interesting to analyse how an individual is acquiring skills related with a task that he/she is not familiar before (i.e. before something is becoming a habitual task) and how awareness contributes in such a situation [2]. For example, someone who is in the mid age but has no prior experience of cycling, it is an interesting phenomenon to find the experiences gone through in the learning process to develop this skill. Most probably at the beginning of this task, it may be really challenging and much attention and awareness are required to perform the task. As a result of that he/she may not be able to change the (visual) focus at all to other environmental cues in order to maintain the balance, keep the ride straight, take turns safely, and pedal up a sloop. Most probably combining what is predicted and what is observed, thereby minimising the error (prediction vs. actual) is the basis for the collected experience in the learning period. Furthermore, how the awareness affects the neural plasticity is an interesting phenomenon too. Although in the learning period, a high level of awareness on the action is important, once it becomes a habitual task such awareness may practically become absent: most probably he/she may not pay any attention at all on pedalling, balancing and keeping straight. Therefore, in daily life experiences predictive and inferential processes for action awareness are important. Nevertheless, when a person learned how to ride a bicycle properly, still he/she may not able to have a small snack or holding an umbrella in a rainy time while cycling (though these can also become habitual tasks for some people). Therefore, just slightly deviating from a habitual task seems to require awareness or new experience.

Moore and Haggard [20] have investigated how predictive or inferential processes of action execution play a role in conscious awareness of an action. They have proposed that awareness of an action is a dynamic combination of both prior awareness (i.e. awareness of the action prediction) and retrospective awareness (i.e. the awareness of the effects of an action), through predictive motor control and inferential sense-making relative to the action execution, respectively (cf. [20, 21]). When a prior awareness state occurs, he/she may become aware of going to perform the action. Having such a prior awareness state still may leave open whether the agent is able to consciously decide to perform or not to perform the action (cf. [1, 4–8]). For example, is still some form of vetoing of the action possible? In principle, the awareness state may play the role of generating a kind of green light for execution of the action. However, equally well the prior awareness state may just play the role of a warning for the agent to be prepared that the action will happen (anyway). As stated in [5, 8] it has been found that for certain types of actions, the decision to perform it is already made at least hundreds of milliseconds (and even up to 10 s) before any awareness state occurs. These findings may suggest that prior awareness often will have no effect on the decision. But this may strongly depend on the type of action. For example, it will be difficult to believe that the action of buying a car or a house remains unconscious and may not be amendable to vetoing based on awareness states (the Monty Hall Problem is also another good situation for this concern). An awareness state can also develop in retrospect, after the action was performed and this will answer the question: ‘*what have I done?*’. Such a retrospective awareness state often relates to acknowledging others from and taking responsibility for having performed the action. It may also play an important role in learning as mentioned in the previous example (i.e. by evaluating the obtained effect in a conscious manner leads to improvement of the performance of the action selection).

This paper extends the work published in [22] by refining the neurologically inspired agent model with more realistic simulation results, new scenarios and a detailed formal specification of the model, together with a more sophisticated parameter estimation methodology. The selected scenarios include a reasonable spectrum of situations in which (a) actions are prepared without being conscious at any point in time, (b) the agent develops prior awareness or retrospective awareness or both. An example of a schizophrenic patient and of a patent in an early stage that may lead to a depression situation are also included. As research questions, this paper mainly contributes to

1. How does the internal prediction process shape or contribute to the (prior) awareness of the action?
2. How does the inferential sense-making shape or contribute to the (retrospective) awareness of the action execution?
3. How does the awareness contribute to action execution?
4. What is the relation and interplay between conscious and unconscious action formation through action ownership and relevant awareness states?

In addition to awareness states, ownership states for an action are also considered in this paper. They are mainly used as important states in the unconscious action formation process. The specific role of the ownership states (in prior and retrospective form) has been separately discussed in [23]; such a more detailed overview is not included in this paper. The structure of the paper organised with a conceptual basis that includes evidences from cognitive neuroscience which followed with a model description in which a detail explanation of the model together with its mathematical basis and formal specification will be presented. To validate the workings of the proposed model, eight scenarios are simulated through a unique parameter value set which was estimated using a systematic approach. Finally, a discussion will be presented highlighting the usefulness of a model in this nature and future works.

## Action awareness viewed neurologically, psychologically and behaviourally

In neurological, psychological and behavioural literature, the notions of awareness and ownership of an action have received much attention. Action ownership is a useful concept which is mainly important to differentiate in how far a person attributes an action to him or herself, or to another person (see [23]). Although in many cases, the feeling that you get when you perform something or another person is performing the same action may be similar, it is clearly possible to identify whether the action belongs to you or to someone else. More importantly, the information about another person’s behaviour influences your self evaluation and vice versa, which makes humans social beings (cf. [24, 25]). After the discovery of mirror neurons, such social phenomena including empathy, imitation, and coordination in a social context can be explained more scientifically as a cognitive process [26–28]. Mirror neurons have been mainly identified in two cortical areas: the posterior part of the inferior frontal cortex and the anterior part of the inferior parietal lobule [28]. They have shown strong correlations not only with specific movements, but also with specific goals (or goal directed actions: e.g. reaching for and grasping an object). From the development perspective of human cognition on self and other representations, their interconnection and how those relate to the cognitive processing were highlighted:

*Over the first several years of life, children acquire knowledge of both objective and subjective aspects of self and others. By 18*–*24* *months of age infants can recognize their own mirror image, a capacity that has been linked to the emergence of self*-*conscious emotions (e.g. embarrassment […]). During the preschool years, children simultaneously develop the capacity to represent their own and others’ mental states […]. This development entails the ability to recognize when self and other perspectives and experiences are shared and thus congruent, and under which circumstances they differ from one another. Interestingly, the development of mental state understanding is functionally related to executive functions […], suggesting that the prefrontal cortex is implicated in self/other cognitive representations. Indeed, neuroimaging data suggest that theory of mind tasks and executive function tasks share overlapping areas of activation in the medial prefrontal cortex* ([24], pp. 527–528)

This separation of self and other is contributing to the ability to recognise ownership of your own action. Research has shown evidence that action prediction (based on sensory information) leads to an action execution with ownership, while when there are problems with action prediction that leads to abnormal states of ownership of that action. For example, when you are tickled by someone, as you have not predicted the action you will experience various sensations due to this sudden action, and the ownership of that action may not be with you (though you do have the body ownership in this situation) (cf. [29]). Chaminade and co-workers [25] have highlighted this:

*The neural underpinnings of internal models for motor control have been investigated with human non*-invasive neuroimaging techniques (for review see [30]). Motor commands that are used by forward models to suppress sensory signals are believed to originate upstream from the primary motor cortex [31], though they may also involve premotor areas in the posterior inferior frontal gyrus [32]. *Actual sensory feedback is used to compute prediction errors for model evaluation and update. When we are tickled by another person * [33] *the sensory consequences of its actions are unpredictable, and the lack of predictability leads to a high prediction error associated with increased activity in the secondary somatosensory cortex. This area, located bilaterally in the parietal opercula* [34], *plays a key role in sensorimotor integration* [35], *and has been involved in the assessment of action ownership* ([25], p. 2, [36])

The nature of human actions varies from direct responses on stimuli to actions that take longer periods to process and react. Here the first types of action are often labelled as automatic or unconscious and the other types as more conscious or intentional [37]. In contrast to action ownership, action awareness is a conscious state. Patients with the anarchic hand syndrome (AHS) [patients with frontal lobe and callosal damage (cf. [38])] always have some form of ownership and awareness of their action but are not able to control the action [39]. For example, in a cafe just seeing a cup of coffee of an unknown person, for an AHS patient might be sufficient to reach and grasp it due to the automatic activation of action plans on this habitual task. In normal context this has been trained as a habitual task, but with the (prior) awareness of people who know when he or she should do this. A few more of such AHS examples are grabbing a doorknob or scribbling with a pencil or combing one’s hair [38]. Furthermore, persons suffering from schizophrenia may easily attribute self-generated actions to (real or imaginary) other persons (see [23]). Furthermore, it has been noted that the problem with AHS patients is to control their action, while with a schizophrenic patient it is a problem with awareness of the action (an AHS patient tries to prevent abnormal behaviour of the alien hand by the good hand after it executed the action) [38]. Through learning with intention and awareness, people have pre-stored actions per stimulus and later without the intention or the awareness the brain will automatically evoke the relevant action which was habitually associated [40]. The frequency and recency of a learned habitual task seem to relate to its probability of getting selected.

The research on action awareness is a challenging task and it is assumed to be that individuals are aware only of the tip of the action iceberg; much further research is necessary to explore and refine the body of knowledge on this (cf. [2, 3, 8, 11, 15, 21]). Nevertheless, there are interesting research findings on this. Empirical evidence collected through an experiment setup proposed by Benjamin Libet and his colleagues [8] has challenged the traditional view of human will and has shown that the brain initiates voluntary movements before we are aware of having decided to move. From a cognitive neuroscience perspective, human actions are mainly a result of signals getting to motor neurons (motoneuron) in the spinal cord mainly via the primary motor cortex and some of its neighbour areas [e.g. pre motor cortex, supplementary motor cortex (SMA)]. Early activation of the primary motor cortex before the agent gets the conscious intention to move (or to act) is called readiness potential and this begins hundreds of milliseconds or even up to 10 s before any awareness state occurs [4, 5, 8]. Therefore, it was proposed that conscious will is an illusion and it is too slow to initiate an action, but action formation is due to an unconscious causal chain of processes and just before the action execution, we will develop the awareness of the action (not as the cause of the action but as an effect of unconscious processes). John-Dylan Haynes has further improved the Libet experiment setup to advance beyond the shortcomings of the experiment (see [5]) and with his findings again the importance of exploring the tightness of the link between unconscious predictive brain processes and subsequent decisions from a conscious perspective is highlighted:

*An important point that needs to be discussed is to what degree the finding of choice*-*predictive information supports any causal relationship between brain activity and the conscious will. Such causal links have been demonstrated previously by direct cortical stimulation over parietal and frontal cortex.* ^*37,80*^ *However, it is unclear if the early predictive signals are also causally involved in the decision. As for the criterion of temporal precedence, there should be no doubt that our data finally demonstrate that brain activity can predict a decision long before it enters awareness. A different point is the criterion of constant connection. A constant connection would require that the decision could be predicted with 100* *% accuracy from prior brain activity. Libet’s original experiments were based on averages, so no statistical assessment can be made about the accuracy with which decisions can be predicted. Our prediction of decisions from brain activity is statistically reliable, but far from perfect. The predictive accuracy of around 60* *% (which is significant, but only 10* *% above chance) can be improved if the decoding is tailored to each subject. However, even under optimal conditions, this is far from 100* *% for several reasons… … …. Importantly, a different interpretation could be that the inaccuracy simply reflects the fact that the early neural processes might only be partially predictive of the outcome of the decision. In this view, even full knowledge of the state of activity of populations of neurons in FPC and in the precuneus might not permit the full prediction of a decision. In that case, the signals have the form of a biasing signal that influences the decision to a degree, but additional influences at later time points might still play a role in shaping the decision. The fact that decoding after the decision from motor cortex can be achieved with higher accuracy might point toward the fact that neural signals in BA10 and in PC are not fully predictive in principle. However, the exact topology of clustering of calls with similar tuning preferences in BA10/PC is, to date, unknown, and thus might turn out to be less suitable for fMRI decoding than in motor cortex* ([5], pp. 16–17)

With the concerns highlighted in the above quote (for more criticisms on this hypothesis see [41]), though the awareness state emerges just before the action execution, it is not yet clear whether there is not at all an impact on action execution from this subjective awareness. One of the issues that have turned out to play an important role both in the execution decisions for an action, and in its attribution, is the prediction of the (expected) effects of the action, based on internal simulation starting from the preparation of the action [42, 43]. If these predicted effects are satisfactory, this may entail a ‘go’ decision for the execution of the action, thus exerting control over action execution. In contrast, less satisfactory predicted effects may lead to a ‘no go’ decision (cf. [44–46]). Predicted action effects also play an important role in attribution of the action to an agent after it has been performed. In neurological research, it has been found that poor predictive capabilities are a basis for false attributions of actions, for example, for patients suffering from schizophrenia [38, 47, 48]. In addition to the predictive effects, the sensation of the actual effect (after executing the action) also has been noted as important in action formation research [15, 17, 21, 37, 43]. In literature, it has been reported that the predicted sensory effect and the sensed actual effect are integrated with each other as a basis for proper attribution of the action [20, 47, 48]. Another element, put forward in [20], is the distinction between action awareness based on prediction (prior to execution), and action awareness based on an inference after execution of the action (in retrospect):

*Our results suggest that both predictive and inferential processes contribute to the conscious awareness of operant action. The relative contribution of each of these processes seems to be context dependent. When we can predict the consequences of our actions, as in a high action*-*effect contingency block, the awareness of action reflects these predictions. This would provide us with a predictive sense of our own agency. In addition, our results show clear evidence that inferential processes also influence the conscious awareness of operant action … … …. The interaction between predictive and inferential processes is of particular interest … … …. The time course over which information about action is built up may be an important clue to this interaction … … …. Sensory feedback provides more precise evidence about actions and their effects. This evidence becomes available only after a short sensory delay, but can then be transferred to memory. Thus, reliable and enduring sensory evidence replaces short*-*lived predictive estimates. We suggest that awareness of action therefore switches from a predictive to an inferential source as the action itself occurs, and as sensory information becomes available.'′*([20], pp. 142–143)

With these evidences, they have suggested that awareness of an action is a dynamic combination of both prior awareness (i.e. awareness of the action-effect prediction) and retrospective awareness (i.e. the awareness of the effects of an action) through predictive motor control and inferential sense-making relative to the action execution, respectively [15, 21, 37, 43] (cf. [19]). Furthermore, Haggard and co-workers presented a new phenomenon called intentional binding: when a voluntary action produces (with the awareness and intention) the temporal (subjective) gap between the action and its perceived sensory outcome is less when the awareness is pre-existing but it is high when the awareness does not involve this [15]. This phenomenon has been argued to be an effect of either prior awareness or retrospective awareness with different experiment setups. To investigate the relation with prior awareness, transcranial magnetic stimulation (TMS) was randomly applied over the motor cortex and the entailed disruption of awareness observed (here through the intention in this setup). A significantly weakened intentional binding has been observed; therefore, it may be useful to highlight the necessity of prior awareness (see [19]). Similarly, there are experiments to analyse the influence from retrospective awareness selecting some tasks where prediction of the action outcome is difficult (or unpredictable) but there is a ‘tone’ after the action execution [20] and it has been observed that retrospective processes play a role when prior predictive processes are absent (or when prediction was minimal). In addition to the mentioned roles in prior and retrospective effects of intentional binding, there are some evidences for its neural basis also:

*Moore and colleagues investigated the contribution of two specific target sites: the pre*-*supplementary motor area (pre*-*SMA) and primary motor cortex (M1). The pre*-*SMA is involved in higher*-*order cognitive aspects of self*-*generated action* [49] *and with the conscious experience of intending to act* [50]. *In this sense it is likely to support predictive contributions to intentional binding. On the other hand, M1 processes signals that are involved in actual motor execution, signals that the authors suggest are required to support inferences of agency … … …. It was found that only stimulation of pre*-*SMA led to a significant reduction in intentional binding. Stimulation of M1 marginally reduced intentional binding, but this effect was not significant. The authors therefore concluded that pre*-*SMA is likely to play a key role in intentional binding.* ([19], pp. 5)

Inhibition and suppressive mechanisms may also be as important as the excitation mechanisms in cognitive control [though some different viewpoints are also put forward (see [51])]. By Gamma-aminobutyric acid (GABA), neurons are performing inhibition at synaptic, circuit and systems levels (cf. [51]). Furthermore, various inhibition types in neuroscience and psychology have been discussed in [51]. Inhibition activates in automatic (e.g. lateral inhibition: if a particular representation accumulates more evidences, that will suppress its fellow representations) and voluntary (e.g. suppression of an irrelevant response, stimulus or memory; in intentionally) manners. Another peculiar aspect that has been observed is that within the process of co-occurrence of predicted effects and sensed actual effects, the predicted effect suppresses the sensed actual effect [29, 52, 53]. Moreover, it has been put forward that the predicted effect and the sensed actual effect are not simply compared or matched, as claimed in the so-called ‘comparator model’ in earlier literature such as [38, 42, 54], but in fact are added to each other in some integration process [20, 47, 48].

Though these evidences are facilitating an adequate level of information to comprehend a theoretical cognitive system in the form of a model, it is further required to confirm these findings; this may have different variants to be explored in future research.

## Description of the cognitive computational model

Having discussed the evidence on awareness (a person’s subjective experience) and ownership (in how far does a person attribute an action to him or herself or to another person) in Sect. 2, this section presents a computational agent model. This model will be used in agent-driven applications where the awareness is paramount (or necessary) for decision-making and justifications of actions through communication. More specifically, this model can provide interesting and important input for problem domains concerning performing or learning specific healthy behaviours or lifestyles. In such domains, having an idea about the extent of the awareness of decisions concerning health or lifestyle is important and the model may provide the fundamentals for applications in these domains (with further refinements and customization where needed). Furthermore, this model may be useful in medical domains where people can analyse and compare the phenomenal effects of certain scenarios through various simulations and further to compare and contrast different hypothesis in theoretical manner to conduct more complex experiments. Also computational simulations have become a promising approach to analyse the emergence in complex systems (e.g. in the aviation domain, or in social science) and a model like this may provide more realistic results especially when human cognitive aspects are necessary in such simulations (e.g. simulating situation awareness in air traffic control, including the human cognitive aspects).

**Fig. 1:**
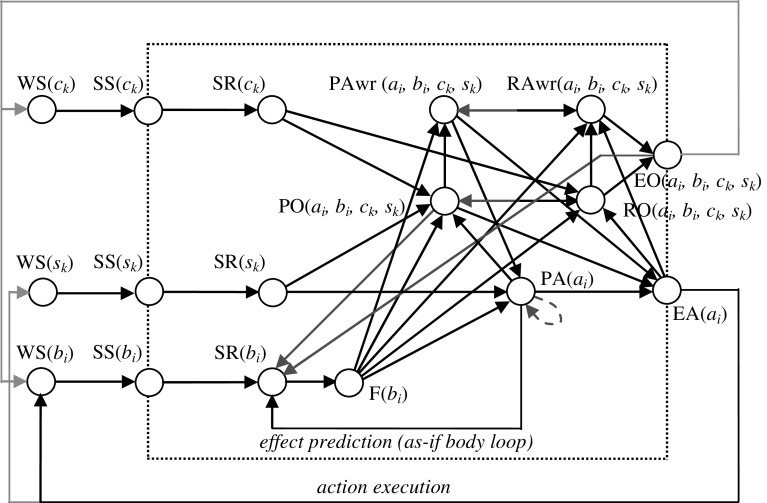
Overview of the computational cognitive agent model. Here an* arrow*  represents a direct activation to state B from state A, an* arrow*  represents a direct suppression to state B from state A, an* arrow*  represents a suppression to all the complements of ‘ith’ state on B_i_ from state A_i_ (where ‘i’ presents an instance of a particular state), and  represents a direct supression to all parellel forms of that state

An overview of the postulated cognitive agent model is presented in Fig. 1 and its abbreviation details can be found in Table 1. The model is a refined version of a previous model presented in [22] but improving the action preparation process and simulation results. In this model, awareness states are taken specific for a given action *a*, effect *b*, context *c* and stimulus *s*. When the context *c* is self, an awareness state for *a*, *b*, *c* and *s* indicates self-attribution awareness, whereas for context *c,* an observed agent B, it indicates awareness of attribution of the action to B. Specific attention is given to ‘self’ than ‘other’ in simulations of this paper (for ‘other’ see [22, 23]). Furthermore, causal relationships in the model are based on the neurological literature presented in the Sect. 2; they do not take specific neurons into consideration but use more abstracted cognitive or mental states for the design of the model (through an interlevel relation between the neurological level and the cognitive/affective mental modelling level). The model uses three world states (WS) as inputs for:

**Table 1:** Nomenclature for Fig. 1

WS(*W*)	World state *W* (*W* can be either: context *c*_*k*_, stimulus *s*_*k*_, or effect *b*_*i*_)
SS(*W*)	Sensor state for *W* (*W* can be either: context *c*_*k*_, stimulus *s*_*k*_, or effect *b*_*i*_)
SR(*W*)	Sensory representation of *W* (*W* can be either: context *c*_*k*_, stimulus *s*_*k*_, or effect *b*_*i*_)
PA(*a*)	Preparation for action *a*_*i*_
F(*b*_*i*_)	Feeling for action *a*_*i*_ after as-if loop or action execution
EA(*a*_*i*_)	Execution of action *a*_*i*_
PO(*a*_*i*_, *b*_*i*_, *c*_*k*_, *s*_*k*_)	Prior ownership state for action *a*_*i*_ with *b*_*i*_, *c*_*k*_, and *s*_*k*_
RO(*a*_*i*_, *b*_*i*_, *c*_*k*_, *s*_*k*_)	Retrospective ownership state for *a*_*i*_ with *b*_*i*_, *c*_*k*_, and *s*_*k*_
PAwr(*a*_*i*_, *b*_*i*_, *c*_*k*_, *s*_*k*_)	Prior-awareness state for action *a*_*i*_ with *b*_*i*_, *c*_*k*_, and *s*_*k*_
RAwr(*a*_*i*_, *b*_*i*_, *c*_*k*_,*s*_*k*_)	Retrospective awareness state for action *a*_*i*_ with *b*_*i*_, *c*_*k*_, and *s*_*k*_
EO(*a*_*i*_, *b*_*i*_, *c*_*k*_, *s*_*k*_)	Communication of ownership and awareness of *a*_*i*_ with *b*_*i*_, *c*_*k*_, and *s*_*k*_

$$ {\text{stimulus}}\,s:{\text{ WS}}\left( s \right) $$

$$ {\text{context }}c:{\text{ WS}}\left( c \right) $$

$$ {\text{effect }}b:{\text{ WS}}\left( b \right) $$

The stimulus *s* represents any internal (bodily, e.g. self-generated facial expression) or external change that may lead to an action execution. Context *c* represents additional information perceived to improve the process of action selection. The context *c* can be differentiated as ‘self’ and ‘other’ (self-other distinction). Effects of mirroring can be modelled when *c* is ‘other’ (see [22, 23]). The effect *b* represents the effects of the execution of an action *a*.

The input world states WS(*s*), WS(*c*) and WS(*b*) lead to sensor states SS(*s*), SS(*c*) and SS(*b*), and subsequently to sensory representation states SR(*s*), SR(*c*) and SR(*b*), respectively. The unconscious causality of action formation has been modelled as explained in [55]: by combining Damasio’s as-if body loop (see [44–46]) and James’s body loop (see [56]) hypotheses. The body loop has been mapped in this model by the following causal relationships:

$$ {\text{WS}}\left( s \right) \to {\text{SS}}\left( s \right) \to {\text{SR}}\left( s \right) \to {\text{PA}}\left( a \right) \to {\text{EA}}\left( a \right) \to {\text{WS}}\left( b \right) \to {\text{SS}}\left( b \right) \to {\text{SR}}\left( b \right) \to {\text{F}}\left( b \right) $$

Damasio extended the body loop concept and argued that the cognitive process of action selection is due to an effect of an internal simulation process prior to the execution of an action [44–46]. The brain will evaluate the effect of each relevant action option [i.e. PA(*a*_*i*_)] by comparing the feelings associated to each individual valuated effects (without actually executing them through the body loop). The simulated option that has the strongest valuated feeling performs as a GO signal through the body loop and else are NO–GO options. The as-if body loop consists of:

$$ sensory \, representation \to preparation \, for \, bodily \, changes \to felt \, emotion $$

In this model, this is represented by the as-if body loop as follows:

$$ {\text{PA}}\left( a \right) \to {\text{SR}}\left( b \right) \to {\text{F}}\left( b \right) $$

The as-if body loop and the body loop demonstrate the working of predicted sensory effects and sensed actual effects, respectively, as highlighted in the Sect. 2. These processes are mainly considered to be unconscious processes involving multiple options for responses evaluated in parallel, to determine an adequate response associated to a stimulus (cf. [40]). Through this parallel internal action simulation mechanism, the agent will not select a random option but the one which has the strongest valuated feeling. Therefore, depending on the weight values attached to each option at that particular moment, the model will show different behaviours in simulations. This purely unconscious mechanism may be interrupted by the effects of awareness (which will be explained later) to select something different by adding some bias to the mentioned process. Being a cyclic process, the effects of an injected bias may have the ability to compete with other options to finally provide a GO signal.

In Fig. 1, state labels are attached with subscript letters *k* and *i*, which indicate, for example, the *k*th instance for a stimulus *s* (e.g. WS(*s*_*k*_)) for a given *s*_*k*_ stimulus and the *i*th option for an action *a* (e.g. PA(*a*_*i*_)). Therefore, through this model, it is possible to have multiple action options either through a single stimulus or from multiple stimuli, depending on the specific model instance.

Each PA(*a*_*i*_) state is affected by its associated feeling through the as-if body loop. Moreover, each PA(*a*_*i*_) state suppresses its complementary options PA(*a*_*j*_) for j ≠ i (as shown in dotted looped red arrow in Fig. 1) proportional to the accumulated strength of that option. This behaviour is in line with the explanation for the lateral inhibition in [51] and will contribute to further strengthen the action selection process. Therefore, naturally the strongest internally satisfied option (which is exceeding a threshold value) will become selected as a result of the unconscious action selection process as explained earlier. The feeling state in this model can be either a positive feeling or a negative feeling. A given stimulus *s*_*k*_ may trigger multiple preparation options in parallel and those might have different associated feelings, also in parallel (note that when a feeling state’s activation level is ‘0’ it is assumed to be a case of no feeling).

Prior ownership states have been integrated with the above-mentioned processes and mainly they get affected from sensory representation states SR(*s*_*k*_) and SR(*c*_*k*_), action preparation state PA(*a*_*i*_) and feeling states F(*b*_*i*_). Also the PO(*a*_*i*_ *, b*_*i*_ *, c*_*k*_ *, s*_*k*_) states affect prior awareness states PAwr(*a*_*i*_ *, b*_*i*_ *, c*_*k*_ *, s*_*k*_), retrospective ownership states RO(*a*_*i*_ *, b*_*i*_ *, c*_*k*_ *, s*_*k*_), action execution states EA(*a*_*i*_) and sensory representation states SR(*b*_*i*_) of effects *b*_*i*_. Having a direct link between SR(*s*_*k*_) and PO(*a*_*i*_ *, b*_*i*_ *, c*_*k*_ *, s*_*k*_) facilitates the embedding of salient features of the input to the ownership and therefore the agent will be able to relate the input and output [together with RO(*a*_*i*_ *, b*_*i*_ *, c*_*k*_ *, s*_*k*_)]. Furthermore, the link from SR(*c*_*k*_) to PO(*a*_*i*_ *, b*_*i*_ *, c*_*k*_ *, s*_*k*_) facilitates the necessary behaviour of mirror neurons when *c* is other (for more details see [23]). The state PO(*a*_*i*_ *, b*_*i*_ *, c*_*k*_ *, s*_*k*_) has a suppressive effect on SR(*b*_*i*_); this provides the mechanism by which the predicted effect suppresses the sensed actual effect (see [29, 52, 53]). Similar to prior ownership, once an action is executed retrospective ownership will develop. The retrospective ownership state RO(*a*_*i*_ *, b*_*i*_ *, c*_*k*_ *, s*_*k*_) is affected by the prior ownership state PO(*a*_*i*_ *, b*_*i*_ *, c*_*k*_ *, s*_*k*_), SR(*c*_*k*_), F(*b*_*i*_) and EA(*a*_*i*_). Furthermore, RO(*a*_*i*_ *, b*_*i*_ *, c*_*k*_ *, s*_*k*_) activation has effects on the states RAwr(*a*_*i*_ *, b*_*i*_ *, c*_*k*_ *, s*_*k*_), PO(*a*_*i*_ *, b*_*i*_ *, c*_*k*_ *, s*_*k*_) and EO(*a*_*i*_ *, b*_*i*_ *, c*_*k*_ *, s*_*k*_). As RO is affected by EA(*a*_*i*_) and F(*b*_*i*_), this provides the cognitive behaviour of retrospective effects as differentiated from prior behaviour. For more details on ownership states of this model, see [23]. Once RO(*a*_*i*_ *, b*_*i*_ *, c*_*k*_ *, s*_*k*_) developed it has a suppressive effect on PO(*a*_*i*_ *, b*_*i*_ *, c*_*k*_ *, s*_*k*_) and through this also it is contributing to the cognitive shift from predictive to inferential.

For each ownership state, an associated awareness state may (or may not) emerge. Awareness states play a higher order cognitive role. The direct links from ownership and feeling to awareness state realise bottom–up activation. Conversely, the effects of awareness states on other states realise top–down activation, which is considered to be a conscious or intended process. Therefore in the presented model, PAwr(*a*_*i*_ *, b*_*i*_ *, c*_*k*_ *, s*_*k*_) is only affected by PO(*a*_*i*_ *, b*_*i*_ *, c*_*k*_ *, s*_*k*_) and F(*b*_*i*_). This is useful to model the idea of Benjamin Libet and others: brains initiate voluntary movements before we are aware of having decided to move (also see the simulations in Sect. 4). Moreover PAwr(*a*_*i*_ *, b*_*i*_ *, c*_*k*_ *, s*_*k*_) affects PA(*a*_*i*_) and EA(*a*_*i*_) and this is reflects the idea of Haggard and co-workers: there may be an impact from this subjective awareness state on action execution. By this PAwr(*a*_*i*_ *, b*_*i*_ *, c*_*k*_ *, s*_*k*_) to PA(*a*_*i*_) link the agent can inject some bias to the current unconscious process through awareness. This may strengthen a weaker action option and improve the predictive feeling of that option (which may lead to getting it executed). Furthermore, in this model PAwr(*a*_*i*_ *, b*_*i*_ *, c*_*k*_ *, s*_*k*_) can also directly strengthen the action execution state. Both the prior ownership states and the prior awareness states are associated to the predictive aspects of the system. In contrast, retrospective awareness states are associated to the inferential aspects, as highlighted in Sect. 2: awareness of an action is a dynamic combination of both prior awareness and retrospective awareness through predictive motor control and inferential sense-making relative to the action execution. Once the RAwr(*a*_*i*_ *, b*_*i*_ *, c*_*k*_ *, s*_*k*_) state is activated, it has a suppressive effect on PAwr(*a*_*i*_ *, b*_*i*_ *, c*_*k*_ *, s*_*k*_) and due to this PAwr(*a*_*i*_ *, b*_*i*_ *, c*_*k*_ *, s*_*k*_) will weaken and RAwr(*a*_*i*_ *, b*_*i*_ *, c*_*k*_ *, s*_*k*_) will be dominant after the action execution. Finally, acknowledging of ownership and awareness of an action is modelled by the connection from the RO(*a*_*i*_ *, b*_*i*_ *, c*_*k*_ *, s*_*k*_) and RAwr(*a*_*i*_ *, b*_*i*_ *, c*_*k*_ *, s*_*k*_) to the EO(*a*_*i*_ *, b*_*i*_ *, c*_*k*_ *, s*_*k*_). Once the state EO(*a*_*i*_ *, b*_*i*_ *, c*_*k*_ *, s*_*k*_) has become activated, it has a suppressive effect on SR(*b*_*i*_) so that this will allow to stop the inferential sense-making process.

In addition to the above mentioned connections a few more suppressive connections are available, which are shown in orange arrows in Fig. 1. These connections are mainly for purposes of having an appropriate scenario. Once a stimulus *s* and context *c* are activated, the agent starts to activate the internal processes as mentioned above and once the agent performed the action, a mechanism is assumed that stops the stimuli as an action effect: the agent has performed the task and due to that environment has changed. Therefore these orange connections: EA(*a*_*i*_) to WS(*s*_*k*_), EO(*a*_*i*_ *, b*_*i*_ *, c*_*k*_ *, s*_*k*_) to WS(*c*_*k*_), and EO(*a*_*i*_ *, b*_*i*_ *, c*_*k*_ *, s*_*k*_) to WS(*b*_*i*_) have been included to stop the input stimulus *s*_*k*_, input context *c*_*k*_ and the effect *b*_*i*_ of action *a*_*i*_. Furthermore, having two inputs (i.e. *s*_1_, *s*_2_, *c*_1_ and *c*_2_) if only one action is executed [let’s say *i* = 1: EA(*a*_1_) and EO(*a*_1_ *, b*_1_ *, c*_1_ *, s*_1_)] then it will be assumed that the executed action will suppress all the inputs, for the purpose of an appropriate scenario.

The following is a brief summary of the agent’s internal causality when given stimulus *s*_*k*_ and context *c*_*k*_ as inputs:

1. *action-effect prediction* sensory representation of effect *b*_*i*_ is affected by preparation of an action *a*_*i*_
2. *preparation for action* *a*_*i*_ is affected by sensory representation of *s*_*k*_, prior-awareness, feeling of effect prediction of action *a*_*i*_ and the complements of current preparation for action *a*_*i*_
3. a *prior ownership state* is triggered based on preparation for action *a*_*i*_, predicted effects *b*_*i*_ of *a*_*i*_, stimulus *s*_*k*_, retrospective ownership and context *c*_*k*_
4. a *prior awareness state* is activated based on feeling of the predicted effect, prior ownership and retrospective awareness
5. *execution of action* *a*_*i*_ is affected by prior-awareness, prior ownership and preparation for action *a*_*i*_
6. a prior ownership state and prior awareness state exert *control over the execution* of a prepared action (go/no–go decision, vetoing)
7. *suppression of the sensory representation of effect* *b*_*i*_ by both prior ownership and communication of ownership and awareness
8. *suppression of the prior ownership state* when the retrospective ownership state is developed
9. *suppression of the prior awareness state* when the retrospective awareness state is developed
10. a *retrospective ownership state* is activated based on co-occurrence of predicted action effects and action effects sensed afterwards
11. a *retrospective awareness state* is activated based on action effects sensed by execution of action *a*_*i*_, retrospective ownership and prior-awareness
12. a retrospective ownership state and retrospective awareness are internal states that also can lead to *acknowledging authorship* of the action (individually), for example, in a social context
13. execution of an action *a*_*i*_ *affects the stimulus* *s*_*k*_ in the world
14. communication of ownership and awareness *affects both context* *c*_*k*_ in the world and *effect* *b*_*i*_ of action *a*_*i*_ in the world

### Dynamics of the model

Connections between state properties (the arrows in Fig. 1) have weights *ω*_*k*_, as indicated in Table 2. In this table, a weight *ω*_*k*_ has a value between −1 and +1 and may depend on the specific context *c*_*k*_, stimulus *s*_*k*_, action *a*_*i*_ and/or effect *b*_*i*_ involved. By varying these connection strengths, different possibilities for the repertoire offered by the model can be realised and can be aligned with the considered scenario and behaviour. Usually weights are assumed to be nonnegative, except for the inhibiting or suppressive connections. The behaviour of the model (through simulations) depends on the values of each of these weights (together with other parameters). Determining proper values for these parameters is a non trivial task.

**Table 2:**
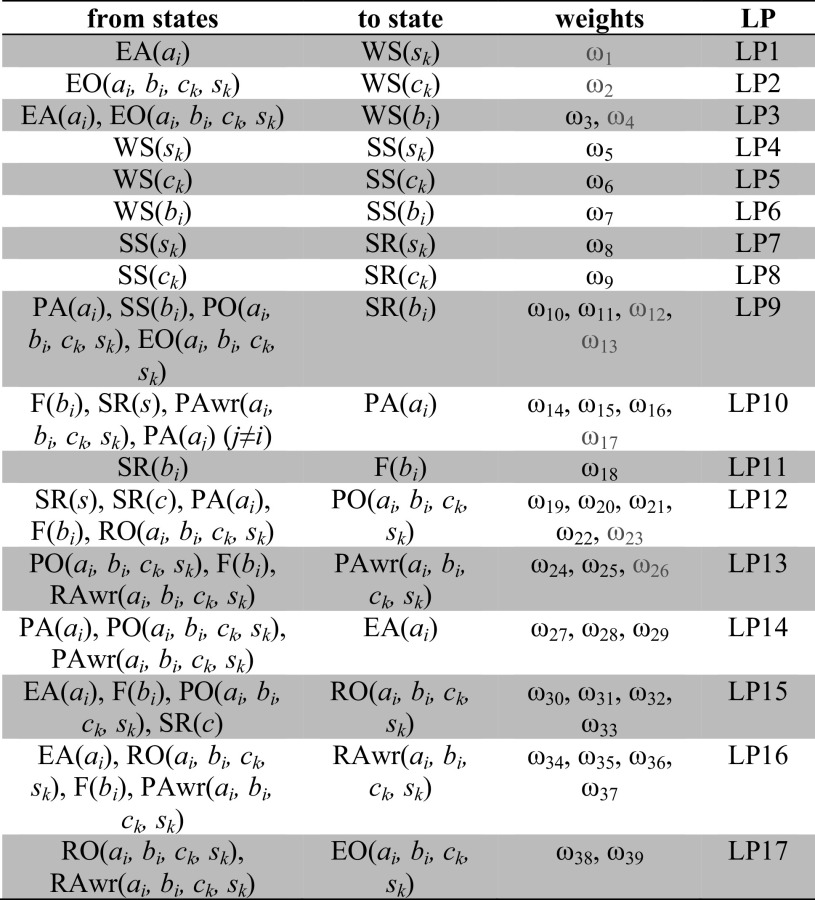
Overview of the connections and their weights

Here the *red colour* ω_k_ indicates negative weights

In this table, the column LP refers to the (temporally) Local Properties: LP1 to LP17 (cf. [57]) and that specifies the update dynamics of the activation value of the ‘to state’ based on the activation levels of the ‘from states’. For the dynamics of each local property, a LEADSTO formalisation is used, which has been shown to be an appropriate approach to model dynamic behaviours of computational cognitive models [57]. LEADSTO is a hybrid modelling language in which a dynamic property or temporal causal relation *a* → *b* denotes that when a state property *a* (or conjunction thereof) occurs, then after a certain time delay, state property *b* will occur (see [57] for the relevance and benefits of LEADSTO in dynamic models). LEADSTO can be compared to Linear Temporal Logic, but differs in the sense that predicate logical state expressions can be used and also real numbers in them. The traces generated for LEADSTO can be seen as continuous time models satisfying the finite variability property: between any two time points there are only a finite number of state changes.

The time delay defined in LEADSTO is taken as a uniform time step Δ*t* here. Table 3 below summarises the formalisation of LP both in LEADSTO format and in differential equation format. This is used as the formalisation of the computational form of the cognitive model described. During the processing, each state property has a strength represented by a real number between 0 and 1 through variables *V* (with subscripts) that run over these values. In dynamic property specifications, this is added as a last argument in the state property expressions. This representation is considered only for the LEADSTO based formalisation. Therefore, the unary predicate representation of each state in the Table 2 was extended to a binary predicate representation by including the state strength *V*_*n*_ [e.g. EA(*a*_*i*_) to EA(*a*_*i*_, *V*_*n*_)]. Furthermore, the temporal step in the original LEADSTO formalisation (see [57]) is used as a uniform time step Δ*t* in this paper. As an example let us consider LP9 according to the LEADSTO formalisation:

**Table 3:**
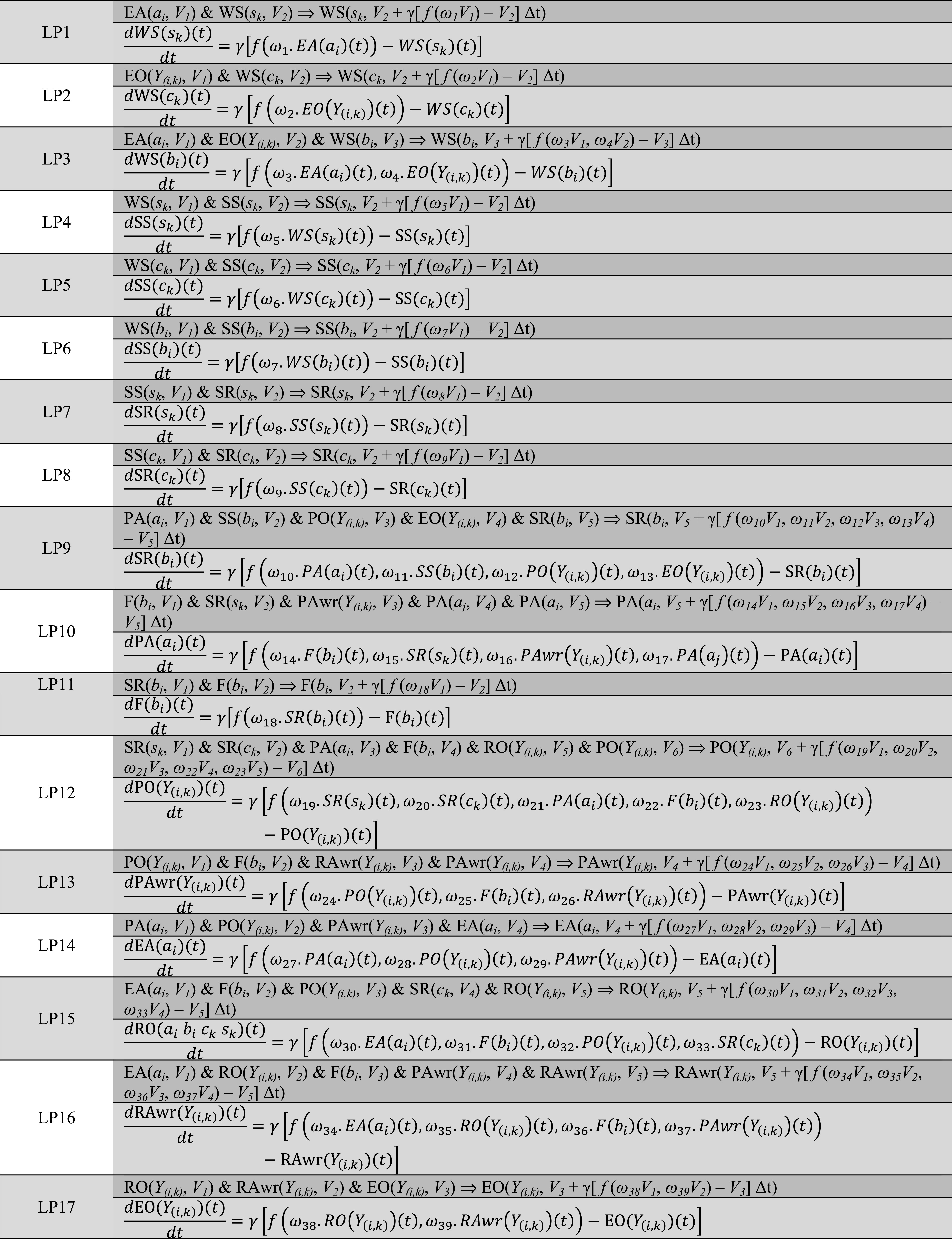
Specification of local properties in the hybrid language LEADSTO and in differential equation format

Here Y_(i,k)_ means (*a*_i_, *b*_i_, *c*_k_, *s*_k_) and for each LP, the first representation is in the LEADSTO and that will be followed by the differential equation format

*LP9 sensory representation for an effect* *b*_*i*_ *state*


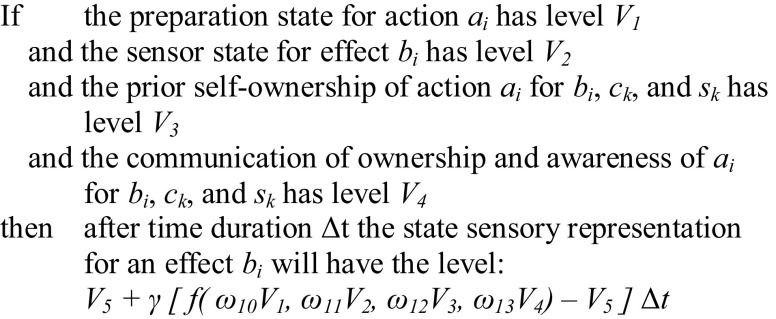


This expresses that after time duration Δ*t*, the value for the sensory representation SR(*b*_*i*_) of effect *b*_*i*_ has changed from *V*_5_ into

$$ V_{5} + \gamma [f\left( {\omega_{10} V_{1} , \, \omega_{11} V_{2} , \, \omega_{12} V_{3} , \, \omega_{13} V_{4} } \right){-}V_{5} ]\Delta t $$

This means that

$$ \Delta {\text{SR}}\left( {b_{i} } \right) = \gamma [f\left( {\omega_{10} V_{1} , \, \omega_{11} V_{2} , \, \omega_{12} V_{3} , \, \omega_{13} V_{4} } \right){-}V_{5} ]\Delta t $$

or

$$ \Delta {\text{SR}}\left( {b_{i} } \right) = \gamma \left[ {f\left( {\omega_{10} .{\text{PA}}\left( {a_{i} } \right), \, \omega_{11} .{\text{SS}}\left( {b_{i} } \right), \, \omega_{12} .{\text{PO}}\left( {a_{i},b_{i},c_{k},s_{k} } \right), \, \omega_{13} .{\text{EO}}\left( {a_{i},b_{i},c_{k},s_{k} } \right)} \right){-}{\text{SR}}(b_{i} )} \right]\Delta t $$

This expression in difference equation format can be rewritten into differential equation format:

$$ \frac{{{\text{dSR}}(b_{i} )(t)}}{{{\text{d}}t}} = \gamma \left[ {f\left( {\begin{array}{*{20}c} {\omega_{10} .{\text{PA}}\left( {a_{i} } \right)\left( t \right),  \omega_{11} .{\text{SS}}\left( {b_{i} } \right)\left( t \right), \omega_{12} .{\text{PO}}\left( {a_{i},b_{i},c_{k},s_{k}} \right)\left( t \right),} \\ { \omega_{13} .{\text{EO}}\left( {a_{i},b_{i},c_{k},s_{k} } \right)\left( t \right)} \\ \end{array} } \right) \quad - {\text{SR}}(b_{i} )(t)} \right] $$

The same formalisation but specifically for the rate of activation change can be presented in its differential form as found in Table 3 under LP9s row. Here, *f* is a function for which different choices can be made. The function *f* should be a combination function (when a given state has only a single input the identity function *f(W)* = *W* is also usable though it is less configurable). For the simulations, the combination function *f* is based on a continuous logistic threshold function *g*(*σ, τ, X*) is used as in the Eqs. (1) and (2).

$$ f\left( {\omega_{1i} y_{1} , \, \omega_{2i} y_{2} , \ldots } \right) = g\left( {\sigma ,\tau ,\mathop \sum \limits_{j } \omega_{ji} y_{j} } \right) $$

with

1 $$ g\left( {\sigma ,\tau ,X} \right) = \left( {\frac{1}{{1 + e^{{ - \sigma \left( {X - \tau } \right)}} }} - \frac{1}{{1 + e^{\sigma \tau } }}} \right)\left( {1 + e^{\sigma \tau } } \right)\quad {\text{when }}x > 0 $$

2 $$ g\left( {\sigma ,\tau ,X} \right) = 0; \quad {\text{when}} \quad X \le 0 $$

In the above equations, *σ* is the steepness and *τ* the threshold; these are configuration parameters that change the shape of the curve and its midpoint on the X-axis. Activation of a state depends on multiple other states that are directly attached to it; therefore incoming activation levels from other states are combined to some aggregated input and perform the activation according to a specification as in LP9 above, or in an alternative differential equation format, as in Eq. (3) [where *g* is the logistic function specified in the Eqs. (1) and (2), and *y*_*i*_ is the activation level of state i].

3 $$ \frac{{{\text{d}}y_{i} }}{{{\text{d}}t}} = \gamma_{i} \left[ {g\left( {\sigma ,\tau ,\mathop \sum \limits_{j } \omega_{ji} y_{j} } \right) - y_{i} } \right] $$

Parameter *γ* is an update speed factor, indicating the speed by which an activation level is updated upon received input from other states. In this model two speed factor values are used: one for the internal states (states which are inside the dotted box in Fig. 1), and the other for the external states: WS(*s*_*k*_), WS(*c*_*k*_), WS(*b*_*i*_), SS(*s*_*k*_), SS(*c*_*k*_), SS(*b*_*i*_), EA(*a*_*i*_) and EO(*a*_*i*_ *, b*_*i*_ *, c*_*k*_ *, s*_*k*_). The internal states’ speed factor is higher than the external states (adhering to the phenomenon that brain neurons are activating much faster than sensor and effector organs).

To obtain a computational specification for temporal simulation of each state, a difference equation is used in the form of Eq. (4).

4 $$ y_{i} \left( {t + \Delta t} \right) = y_{i} \left( t \right) + \gamma_{i} \left[ {g\left( {\sigma ,\tau ,\mathop \sum \limits_{j \in \,s\left( i \right)} \omega_{ji} y_{j} } \right) - y_{i} \left( t \right)} \right]\Delta t $$

By having different values for each parameter (i.e. for weight values *ω*_*i*_, time step size Δ*t*, slow and fast speed factors *γ*, steepness *σ*_*i*_, threshold *τ*_*i*_), the agent can facilitate a wide variety of behaviours. Each LP in Table 3 is represented in a computational form (by the JAVA language) and the dynamics of the system is achieved through evaluating the causality effects through a set of difference equations as in Eq. (4). For each discrete time step Δ*t* the behaviour of each state is calculated the emergence of the behaviour is traced with a identified parameter value set. From a mathematical point of view, the dynamics of the model is (numerically) solving the differential equations of LP1 to LP17 by assuming that at time *t* = 0, WS(*s*) and WS(*c*) holds value 1 as state level.

Some of these behaviours are presented as simulations in Sect. 4.

## Simulation results

In this section, simulation experiments for a number of example scenarios are discussed, which all involve the occurrence of a preparation state for an action *a*_*i*_, triggered by some stimulus *s*_*k*_ and context *c*_*k*_. These scenarios relate to phenomena in the literature, as discussed in Sects. 1 and 2. They have been generated based on the specification presented in Sect. 3. Eight scenarios have been simulated to highlight the different possible behaviours of the model and among those three scenarios are new, whereas the other five were selected from the previous work in [22] but those behaviours have been significantly improved in the newer versions presented here. Furthermore, for the scope of this paper only *c* is ‘self’ situations are selected (for some examples where *c* is ‘other’ see [22, 23]). The following is a summary of the different simulated scenarios:

1. The first scenario simulated describes a situation where the prepared action has satisfactory predicted effects and therefore is executed; in this case both prior and retrospective awareness states occur. This scenario will be considered as the base case for the interplay between conscious and unconscious processes.
2. The second scenario simulated describes a situation where the prepared action has satisfactory predicted effects and therefore is executed but the awareness is absent (in other words merely an unconsciousness action). The strength of the action execution is lower as compared to the first scenario.
3. The third scenario simulated describes a situation where the prepared action lacks satisfactory predicted effects, and is therefore not executed: a no–go decision, or vetoing in unconscious form. Furthermore, the awareness state is almost absent due to the almost absent feeling.
4. The fourth scenario simulated describes a situation where a poor action prediction capability is modelled: the action effect is falsely predicted as satisfactory. This leads to a prior ownership state, which is sufficient to actually execute the prepared action. In this case, a low retrospective ownership state and almost absent retrospective awareness state will occur, as the sensory representation of the effect stays low. This simulation is used to explain the basic cognition and behaviour of a schizophrenic patient.
5. The fifth scenario simulated describes a situation where two prepared actions exist for two input stimuli (*s*_1_, *s*_2_, *c*_1_ and *c*_2_) but one is relatively less positive compared to the other on predicted effects (difference is 0.2 in terms of the weight *ω*_10_). The one which is less positive (2nd option) gets diluted over time in terms of PA(*a*_2_), SR(*b*_2_), F(*b*_2_), PO(*a*_2_, *b*_2_, *c*_2_, *s*_2_) and PAwr(*a*_2_, *b*_2_, *c*_2_, *s*_2_) while the other prepared action gets executed and develops the retrospective awareness too.
6. The sixth scenario simulated describes a situation exactly as in the fifth scenario but in this case once the action with the strongest predictive effect [i.e. SR(*b*_1_)] is executed it does not suppress the inputs *s*_2_ and *c*_2_. Therefore, once EA(*a*_1_) is executed because of the existence of *s*_2_ and *c*_2_ the agent is preparing for EA(*a*_2_) and successfully this will be performed. This confirms the agent capacity of cognitive control combining both conscious and unconscious processes.
7. The seventh scenario simulated describes a situation where the agent is prepared for an action by expecting a particular effect *b*_1_, though it is actual effect after execution is different: *b*_2_ (mismatch between the predicted and actual). As claimed by Haggard, this scenario contributes to the idea that awareness of an action is a dynamic combination of both predictive and inferential sense-making. Having a strong predicted effect agent develops a strong prior awareness, but not sensing the same effect it leads to a poor retrospective awareness of that predicted effect. This phenomenon is important for the agent’s learning process through error correction.
8. The eighth scenario simulated describes a situation that can be considered as an early stage of a cognitive impairment for depressive symptoms. In this case, the agent is preparing for two action options where one is having a positive feeling while the other is with a negative feeling [i.e. F(*b*_1_) and F(*b*_2_)]. According to the biased nature on negative feelings, the agent consciously affects selection of the negative one, though both options are identical from an action selection perspective (i.e. by having exactly the same values for all the parameters for each option except for *ω*_29_). Due to this conscious biased influence agent will execute the action with a negative feeling and it is considered to be that repeatedly performing these type of thoughts/tasks it will develop a negative mood that leads to a depression situation (in a long run).

Although these simulations will be presented in more abstract manner without relating them to real examples all the time, in general from a real-world perspective, the following example explanation of a scenario can be kept in mind:

- Stimulus *s* is that you need to withdraw some money.
- Context *c* is that you are in a bank and in front of an ATM.
- Action *a* is clicking on specific buttons to withdraw € 55.
- Effect *b* of action *a* is that you get € 55 cash.
  This simple happy example scenario may have many variations such as:
- the person may doubt whether to directly withdraw € 55 or to first check the current balance in the account
- once the person has entered € 55 to withdraw, the system may inform the person that it does not have € 5 notes and ask to change the amount
- may be surprisingly ATM will return the card but without the money

Furthermore, when considering complex systems like Air Traffic Management there will be many examples that this model can be utilised (see [58] for more examples).

### Selecting values for parameters of the model

Selecting parameter values for a dynamic cognitive model which consists with set of differential equations is a nontrivial research challenge. This will be even more difficult when there are no detailed numerical empirical data to use in this process, but only some characteristics of behaviours available in more fuzzy form (cf. [59]). Furthermore, another problem with computational cognitive models is that for different types of persons with different behaviours it may seem necessary to find a unique person-dependent set of parameters from scratch. As an alternative, if it is possible to identify a particular parameter value set which is able to demonstrate a variety of situations using only very minimum number of variations this would make the issue easier to handle. Therefore, in this work the focus has been on finding such a generic parameter value set that provides more confidence from the model validation perspective and its practical usage in future complex applications. The current model consists of many parameters: 39 weight values (for one option: *k* = *i* = 1): *ω*_*i*_, a time step size: Δ*t*, slow and fast speed factors: *γ*, 17 steepness: *σ*_*i*_, and 17 threshold: *τ*_*i*_. Table 4 presents connection weight values and Table 5 presents threshold (*τ*) and steepness (*σ*) values used in configurations of simulations on this cognitive model. From these it is clear that the weight value set is generic and just changing very few weights (in most of the cases either one or two) have obtained the different expected behaviours.

**Table 4:**
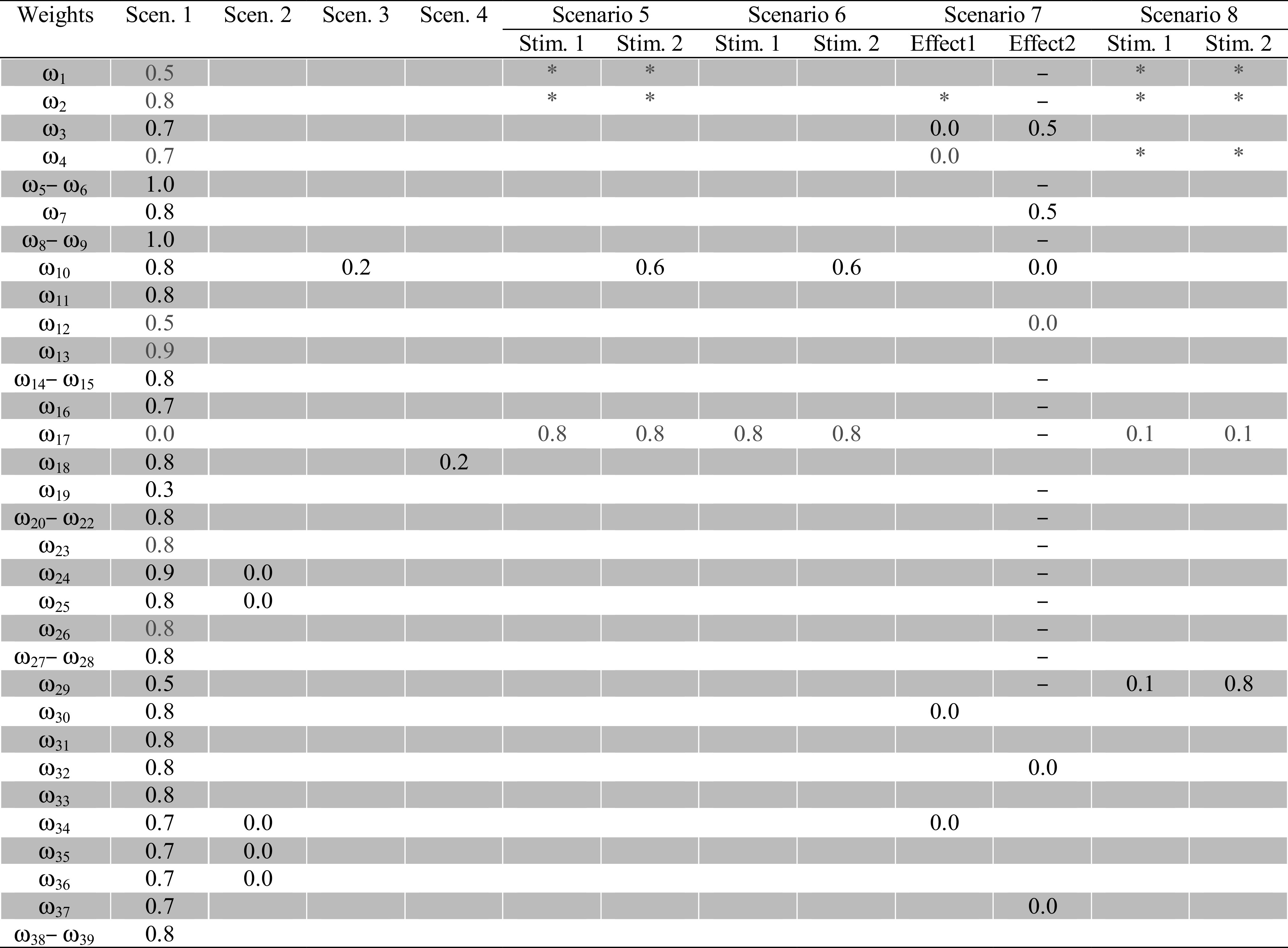
Connection weight values used for cognitive agent model

In here if a value of a particular weight is empty for a scenario that means it is equal to the value of that in the Scenario 1. Furthermore if a value is ‘–’ then such a link was not existed for that scenario and furthermore ‘*’ presents that the particular link suppresses both its mapping inputs and all the remaining

**Table 5:** Threshold (τ) and Steepness (σ) values used in configurations of simulations

	WS(*s*_k_)	WS(*c*_k_)	WS(*b*_i_)	SS(*s*_k_)	SS(c_k_)	SS(*b*_i_)	SR(s_k_)	SR(*c*_k_)	SR(*b*_i_)
σ	1.00	1.00	6.00	2.00	2.00	3.50	2.00	2.00	3.00
τ	0.01	0.01	0.01	0.01	0.01	0.01	0.01	0.01	0.01

	PA(*a*_i_)	F(*b*_i_)	PO(*Y*_(i,k)_)	PAwr(*Y*_(i,k)_)	EA(*a*_i_)	RO(*Y*_(i,k)_)	RAwr(*Y*_(i,k)_)	EO(*Y*_(i,k)_)	
σ	2.00	3.50	6.00	5.00	6.00	5.00	4.00	5.00	
τ	0.50	0.01	1.00	0.60	1.00	1.90	1.60	0.80	

The main challenge in this approach is that there is no real detailed data value set that can be compared to the output of the agent model to estimate parameters. Only certain features of the behaviour of each cognitive state are known for different scenarios, based on neurological and behavioural evidence from the literature (for example prior awareness should occur before the action execution and after prior ownership, there should be a dip in the sensory representation in-between predictive representation and inferential representation, et cetera). To identify the parameter values a systematic approach is used. For this approach, it is a necessary condition to select multiple scenarios (minimum is three but having more will improve the quality of the results) which are interrelated from a functional point of view. For example, the first scenario is considered to be as a reference scenario and the second scenario is different from that just by achieving that awareness states are not developing (i.e. *ω*_24_ = *ω*_25_ = *ω*_34_ = *ω*_35_ = *ω*_36_ = 0). Furthermore, the third scenario handles a case in which a prepared action lacks satisfactory predictive effects (i.e. *ω*_24_ = 0.2), and the fourth scenario addresses poor action prediction capability (i.e. *ω*_18_ = 0.2); and so on (see Table 4). This interrelation among scenarios is very important for a minimum number of parameter changes enabling to identify a generic parameter value set for the model.

In this parameter estimation approach the idea is as follows:

- First scenario is addressed and parameter values are calibrated to simulate its behaviour as identified through the literature.
- Then using the obtained parameter value set, by changing just a few (scenario-related) weight values it is checked whether the model with these parameter settings is able to generate the behaviour for the second scenario.
- If this provides a simulation with a pattern as expected (without changes to the previously obtained parameter values, except for the changes particular to the current scenario) then it provides a good confidence on the current identified parameter value set,
- But if not, then it is necessary to change the parameter values of the first simulation (based on the sensitivity of certain parameters on the required final output) until the behaviours for both simulations are satisfactory.
- This approach is incrementally extended to each scenario until a generic parameter value set for all the scenarios has been identified. For any new scenario if any changes to the previously obtained parameter values are required, then all previously addressed scenarios are readdressed.
- In the first few iterations, it may challenging to identify a parameter value set, but over time the convergence is really fast and it will be experienced that the identified parameter value set is more generic and it is facilitating the necessary behaviours for simulations even without any changes to the obtained parameter value set.

In addition to the parameter values in Tables 4 and 5 for the step size (Δ*t*), slow speed factor (*γ*), and fast speed factor (*γ*) parameter values 0.25, 0.6 and 0.7 were used respectively for all the scenarios.

### Scenario 1: normal execution with ownership and awareness

The first scenario considered describes a situation where the context *c* is the agent itself, and a stimulus *s* occurs. The action effect *b* of *a* is considered positive for the agent and the awareness of action formation and execution will occur, together with generated prior and retrospective ownership states. The following execution trace will be expected from the agent here:

- External stimulus *s* and context *c* will occur and trigger preparation of action *a*.
- Based on the preparation state for *a,* the sensory representation of the (positive) predicted effect *b* of *a* is generated.
- Based on this positive predicted effect and the other states, a prior ownership state for action *a* is generated.
- Prior ownership for action *a* is followed by the prior awareness; this is generated just before the action execution.
- The prior ownership and prior awareness states for action *a* lead to actual execution of action *a*.
- The execution of *a* affects *b* in a positive manner and propagates to the sensory representation of *b* and the feeling of *b*.
- At the same time the sensory representation of *b* is suppressed due to the prior self ownership state.
- Based on the generated states, after the execution of action *a* the agent develops a retrospective ownership state.
- Retrospective ownership of sensed effect *b* of action *a* is followed by retrospective awareness of action *a* and its effect
- Finally, the agent communicates this ownership and awareness of it.

**Fig. 2:**
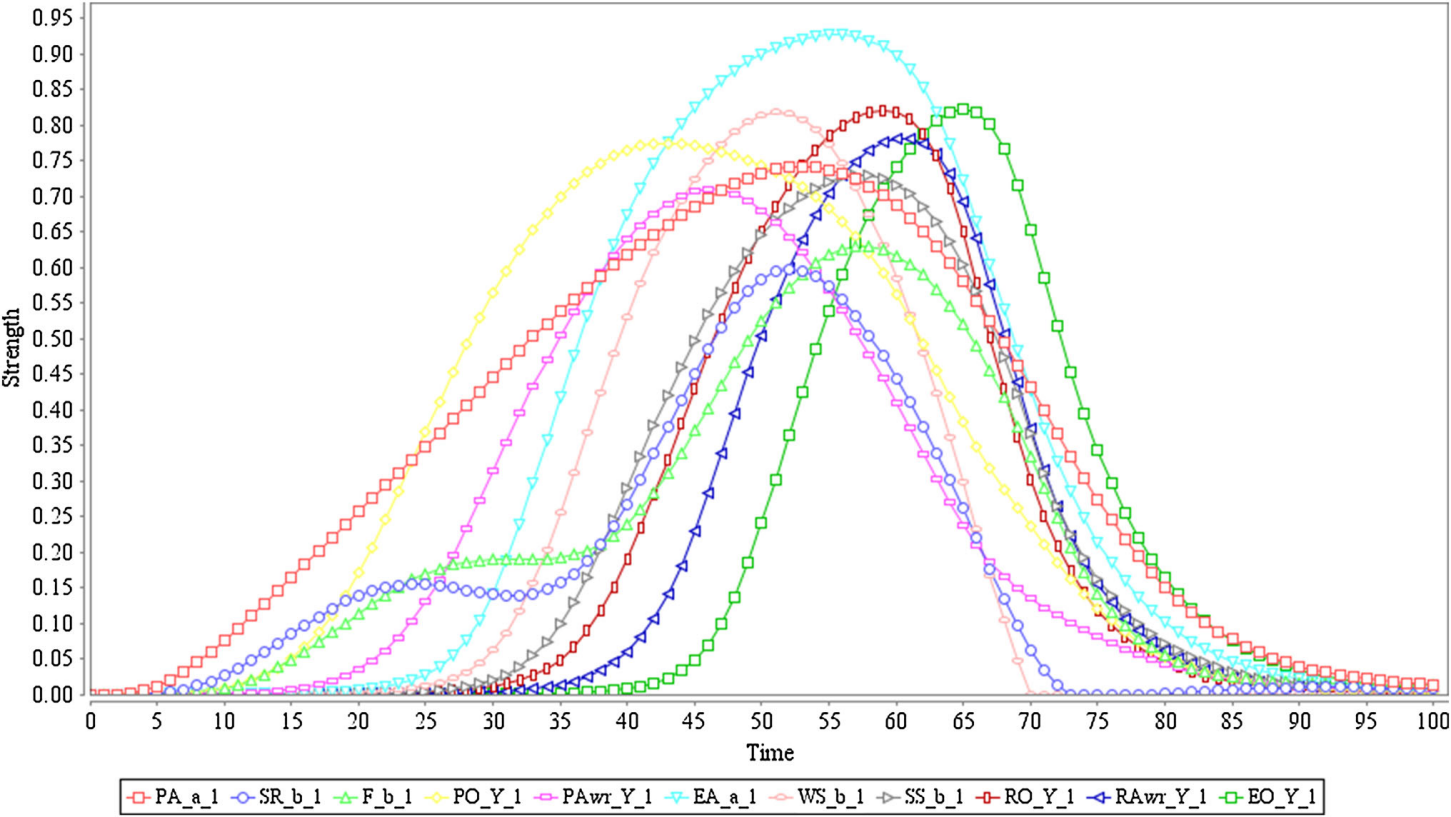
Scenario 1: Executing an action with ownership and awareness. In here ‘Y_1’ represents ‘*a*_1_, *b*_1_, *c*_1_, *s*_1_’

The simulation result of this scenario is shown in Fig. 2. In this figure, it is shown that (after sensing the stimulus) the agent triggers preparation of action *a* from time point 3 on (with a peak value of 0.74 around time point 55). Based on that the sensory representation of the predicted effect *b* of *a* is generated (through the as-if body loop with peak value 0.15 around time point 15 and through the body loop with peak value 0.59 after action execution around time point 50). This is followed by the feeling of *b* (through the as-if body loop with the peak value 0.19 and through the body loop with the peak value 0.62). These states contribute to the generation of a prior ownership state which starts to trigger at time point 5 and reaches a peak value of 0.77 around time point 57. After activating prior ownership, prior awareness is developing, mainly upon the formation process of effect prediction *b* of *a*, and its associated feeling. The prior awareness has started to pop up around time point 13 and has obtained peak value 0.71 around time point 45. As a result of the prior ownership and awareness states, the agent initiates the actual execution of action *a* which propagates its effects through the (external) body loop. This clearly shows that prior awareness is just before the action execution (cf. [4, 5, 8]) as the action execution process started at time point 15, and has its peak around time point 55, with maximum strength 0.92. Furthermore, it shows that via the body loop and the sensor state, the execution of action *a* also affects the sensory representation of *b* and the feeling of *b*. Therefore, the sensory representation *b* of *a* behaves as expected, by adding the sensed actual effect to the predicted effect, and the same effect is propagated to the feeling of *b* too (cf. [20, 47, 48]). Due to the action execution, the agent develops a retrospective ownership state (starts at time point 26 with peak value 0.82 around time point 58), which is followed by a retrospective awareness state (starts at time point 29 with peak value 0.78 around time point 62). Finally, the agent communicates ownership and awareness of it for the performed action, based on the retrospective awareness and ownership states (with the maximum strength of 0.82 around time point 67). Note that when the stimulus is taken away (as explained in Sect. 3 this has been performed through an external suppressive mechanism through the orange colour arrows in Fig. 1), all activation levels will come down to 0; they will come up again when a new stimulus occurs. Note that the numerical information related to the time scale or the peak values has not been coupled with actual brain signals but is only used as a frame of reference.

### Scenario 2: normal execution with ownership but without awareness

There are many situations in which human action formation occurs merely unconsciously, especially when related to habitual tasks [40]. As in the first scenario above also in this case, the agent will experience that the prepared action has satisfactory predicted effects. Nevertheless, the agent will not develop any awareness state of the experienced feeling. The following execution trace will be expected from the agent for this scenario.

- External stimulus *s* and context *c* will occur and trigger preparation of action *a*.
- Based on the preparation state for *a,* the sensory representation of a (positive) predicted effect *b* of *a* is generated.
- Based on this positive predicted effect and the other states a prior ownership state for action *a* is generated, but no prior awareness
- The prior ownership state for action *a* leads to actual execution of action *a*.
- The execution of *a* affects (via sensing) the sensory representation of *b* and the feeling of *b* in a positive manner
- At the same time, the sensory representation of *b* is suppressed due to the prior ownership state.
- Based on the generated states, after the execution of action *a*, the agent develops a retrospective ownership state, but no retrospective awareness.
- The agent does not communicate this ownership.

**Fig. 3:**
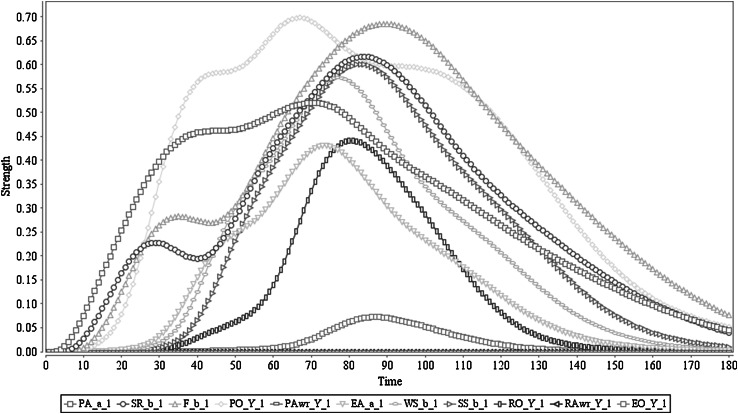
Scenario 2: Executing an action with ownership and no awareness. In here ‘Y_1’ represents ‘*a*_1_,* b*_1_,* c*_1_,* s*_1_’

The simulation result for this process is presented in Fig. 3. The agent starts to prepare for action *a* at time point 2, and for this preparation a peak value of 0.52 is obtained. Together with the action preparation, agent develops the sensory representation of predicted effect *b* of *a* (with peak value 0.15 based on the as-if body loop, and peak value 0.61 through the body loop) and the associated feeling of *b* (with peak value 0.68). Based on this predictive information, the agent develops prior ownership from time point 6 on and with peak value 0.7. More importantly, in this simulation prior awareness has not developed. The developed states lead to performing the action *a* which starts at the time point 14 and obtains peak value 0.43. The execution positively affects (via the sensor state) the sensory representation of *b* and the feeling of *b* (adding the sensed actual effect to the predicted effect). With this action execution effect, the agent develops retrospective ownership with peak value 0.44. Finally, the agent does not communicate the ownership about the performed action (has a very low strength due to lack of awareness).

This cognitive behaviour trace is in line with the expectations, and mainly when comparing it to Scenario 1, it demonstrates the possible impact of the awareness states. For example, when a prior awareness state occurs, it facilitates an enhancing effect on action preparation, sensory representation and feeling and also provides much smoother effects (as seen in Fig. 2). Nevertheless, in this Scenario 2, these states have lower activation levels (also the action preparation state), that may be attributed to the absence of enhancing effects through prior awareness. Also this scenario highlights the strength of action execution with awareness: the action has executed with a relatively high peak value (0.92 vs. 0.43). This might be explained as an influence from prior awareness to action execution. Nevertheless, according to the literature, further research is required to conclude this; cf. [5]. From the computational perspective, this at least confirms the model’s capability of action formation both with awareness and without awareness.

### Scenario 3: prepared action lacks satisfactory predicted effects

Humans are not always responsive to all the environmental stimuli (even in unconscious form). Nevertheless, there should be an explanation from the perspective of internal processes when receiving a stimulus why that stimulus does not lead to an actual action execution. This simulation provides the behaviour for such situation and explains how the lack of (positive) predicted effects generated through the as-if body loop relates to this. The following execution trace will be expected for this scenario:

- External stimulus *s* and context *c* occur and trigger preparation of action *a*.
- Based on the preparation state for *a,* only a weak sensory representation of predicted effect *b* of *a* is generated.
- Poor predictive effects will be reflected through the feeling state with very low activation.
- Based on this poor predicted effect *b* and the other states, a low prior ownership state for action *a* is generated.
- Due to the poor prior ownership and low predictive feeling states, the agent does not develop adequate prior awareness.
- The low prior ownership state for *a* does not lead to actual execution of action *a*; the action *a* can be considered vetoed
- The agent develops no retrospective ownership state for *a* and no retrospective awareness.
- The agent does not communicate ownership or awareness.

**Fig. 4:**
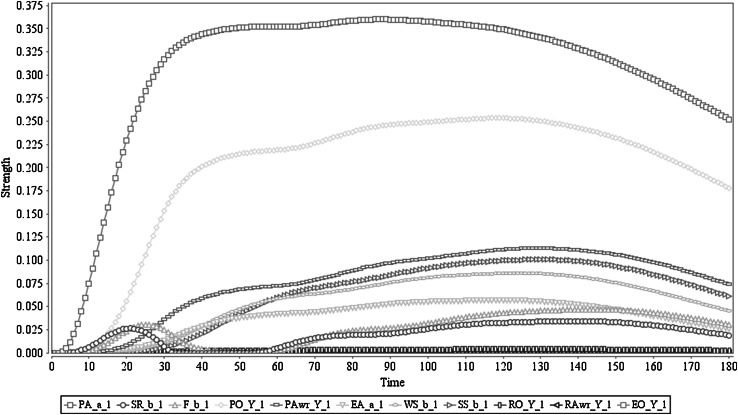
Scenario 3: Prepared action lacks satisfactory predicted effects. In here ‘Y_1’ represents ‘*a*_1_,* b*_1_,* c*_1_,* s*_1_’

The simulation of this scenario is shown in Fig. 4. The predicted effect is very low compared to the Scenario 1. This clearly shows that the action *a* triggered by stimulus *s* (which has an effect *b*) is not positive for the agent (in other words it is more like neutral to the agent in terms of the feeling): it leads to not getting any feelings out of it. Nevertheless, the prediction capabilities are assumed correct in this case, so no high level of *b* is correctly predicted for *a*. As a result of this low prediction, the prior ownership state also stays at a very low level. Due to this, prior awareness is not developed (stays in a very low level), which would be needed to strengthen the action execution. Therefore execution of the action also stays very low (below 0.1) and due to that, there is no retrospective ownership state, nor communication of ownership. Having a single weight value change [*ω*_10_: PA(*a*_*i*_) to SR(*b*_*i*_) from 0.8 to 0.2] to obtain this behaviour from the reference Scenario 1 shows the coherent nature of action formation and higher order coupling as a process. This shows, from a complex systems simulation perspective, how the same agent model by limited variations in assigned parameter values demonstrates qualitatively different results. Furthermore, this confirms that the model has adequately adapted Damasio’s hypothesis: in the agent’s decision-making process, it has to assess the incentive value of the choices through an internal simulation process.

### Scenario 4: poor feelings of action prediction effects of a schizophrenic patient

In the previous scenario, it was presented how the lack of satisfactory predicted effects will lead to a No–Go decision on action execution. In the current simulation scenario, the focus is on poor feelings of predicted effects (the satisfaction level of the predicted effect is high but it does not adequately feel to the agent). In this situation the action effect *b* for action *a*, in principle is positive for the agent, like in the first scenario. Nevertheless, the agent will not properly feel the effects of prediction. This is what assumed to happen in (at least some) patients with Schizophrenia [38, 47, 48]. Much evidence exists that relates this to poor emotional aspects in expression, experience and perception (mainly due to abnormalities in the workings of Amygdala) [60, 61]. Schizophrenic patients often have the impression that their own actions are being created, not by themselves, but by someone from the outside [38]. The following execution trace will be expected for such a phenomenon:

- External stimulus *s* and context *c* occur and trigger preparation of action *a*.
- Based on the preparation state for *a* the sensory representation of predicted effect *b* of *a* is generated.
- Lack of feeling of predicted effects will be experienced.
- Based on this predicted effects and its poor feeling, a relatively low level of a prior ownership state for action *a* is generated.
- Based on this low level of prior ownership and poor feelings, a relatively low level of prior awareness will be developed.
- This prior ownership and awareness levels for action *a* are still sufficient to lead to actual execution of action *a*.
- The execution of *a* affects *b* in a positive manner and (via sensing) the sensory representation of *b* but still the felt feelings are weaker.
- Due to poor feelings, agent will not develop adequate level of retrospective ownership and retrospective awareness.
- The agent does not communicate ownership or awareness for action *a*.

**Fig. 5:**
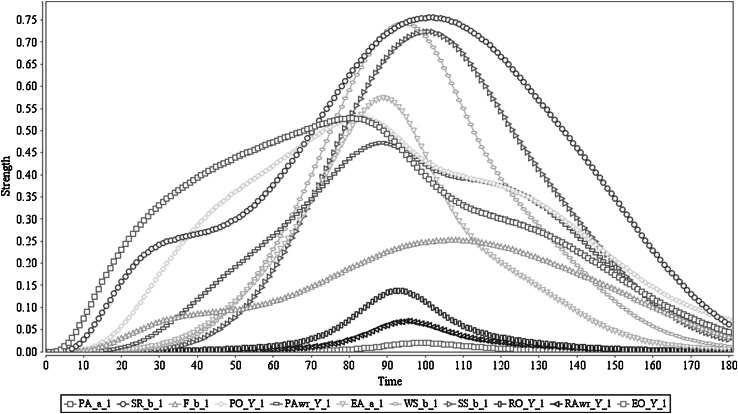
Scenario 4: Poor feelings of action prediction effects of a schizophrenic patient. In here ‘Y_1’ represents ‘*a*_1_,* b*_1_,* c*_1_,* s*_1_’

The simulation of this scenario is shown in Fig. 5. In this case, the agent has not fully felt the predicted effects of action *a*. After sensing, the stimulus agent has triggered preparation of action *a* at time point 3 (with a peak value of 0.52). Based on that the sensory representation of predicted effect *b* of *a* is generated (through the as-if body loop with peak value 0.25 and through the body loop with peak value 0.59) and followed by the feeling of *b* (through the as-if body loop with peak value 0.08 and through the body loop with peak value 0.25). This clearly shows that the predicted effect has not been properly felt by the agent due to very low values for feeling state F(*b*_*i*_). Next these states contribute to generate a prior ownership which starts to trigger at the time point 6 with peak value 0.52. Together with the prior ownership, the agent has experienced prior awareness with peak value 0.47 (this strength is relatively less compared to the same in the first scenario: 0.71). The prior ownership and awareness levels are much better in this case compared to the situation in the third scenario. Therefore, in contrast to the third scenario, these levels turn out high enough for the execution of the action. The maximum strength of the actual execution of action *a* is 0.57 and this execution has positive effects which are sensed. Therefore, the sensory representation *b* of *a* behaves as expected after adding the sensed actual effect to the predicted effect. Nevertheless, the agent has again not properly felt the effects: it has not developed a sufficient strength for the feeling state. Due to these poor perceived effects of feeling, the agent has not developed an adequate level of retrospective ownership and no retrospective awareness. This behaviour can be interpreted as having some strength for the prior ownership and awareness for the action but no retrospective values for it. The agent may reach an internal conflict situation where the action seems not being created by itself, but by someone else. From the parameter values’ perspective, this result has been achieved only with a single change from the first scenario on *ω*_18_ [from SR(*b*_*i*_) to F(*b*_*i*_)] from 0.8 to 0.2. Nevertheless, the cognitive impairment behind a schizophrenic patient is more complicated than modelled here; for example, the impact of some other states (perception, attention, emotions, etc.) has to be considered as well. Therefore, it is required to extend the current model to provide more realistic cognitive behaviour for Schizophrenia. Nevertheless, the current behaviour already demonstrates some of the the basics.

### Scenario 5: cognitive controlling when multiple action options compete

**Fig. 6:**
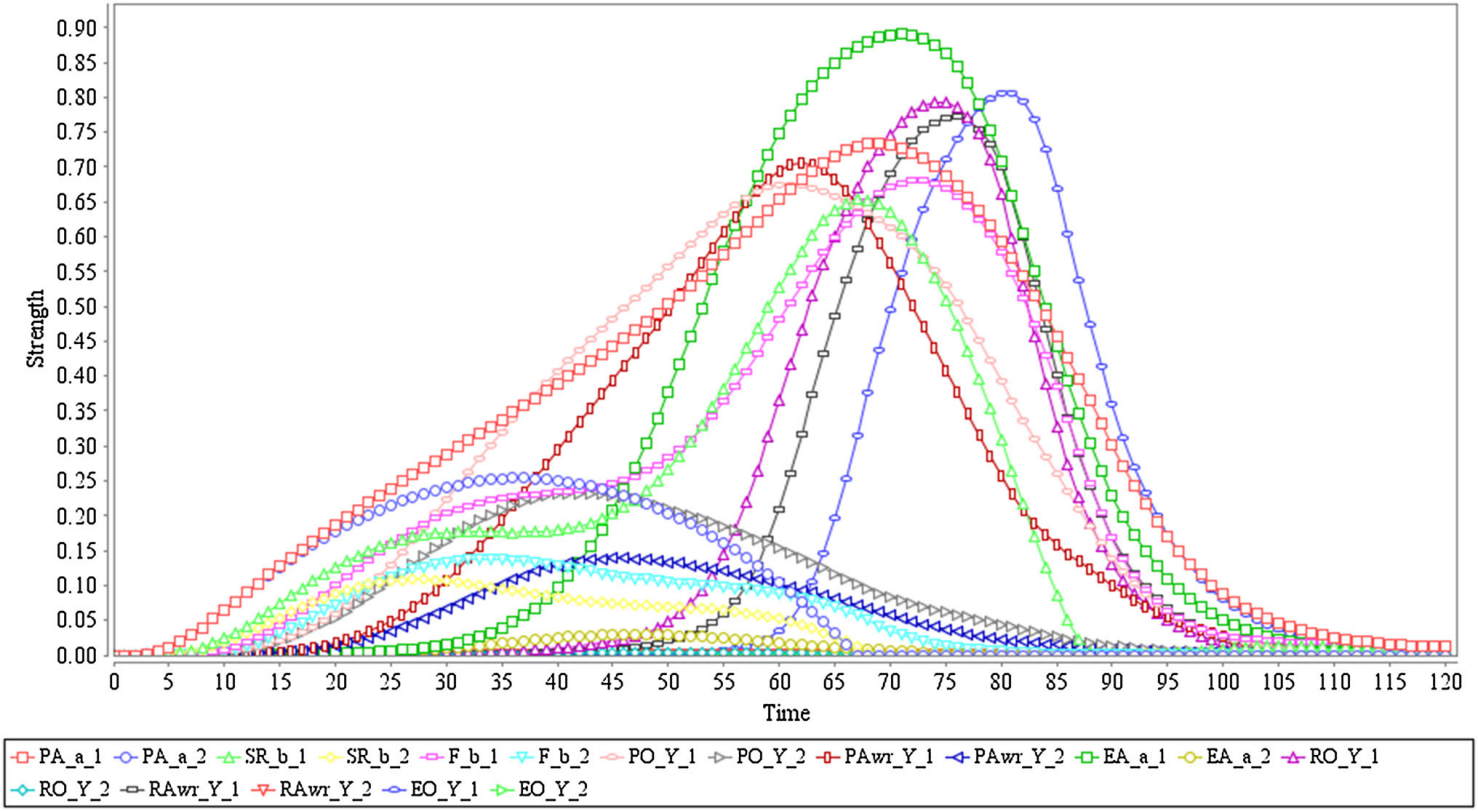
Scenario 5: Cognitive controlling when multiple action options compete. In here ‘Y_1’ represents ‘*a*_1_,* b*_1_,* c*_1_,* s*_1_’ whereas ‘Y_2’ represents ‘*a*_2_,* b*_2_,* c*_2_,* s*_2_’

The fifth simulation scenario explains a situation when two action options exist (for the simulation two input stimuli were considered for this) and both have the potential to execute an action. The competition among the two options and the cognitive control through unconscious and conscious processes on this action selection is captured, resulting in execution of only one of the actions. For this behaviour two independent input tuples (*s*_*k*_ *, c*_*k*_) were used, occurring in parallel (nevertheless it is possible to use a single input that triggers two action options, but due to the requirements in the next scenario the mentioned approach was used). For each option, the same configurations were used as in the first scenario, except for few weights (see Table 4). In this scenario, for the connection strength related with option 2 (i.e. *k* = *i* = 2) from the action preparation *a*_2_ to its predicted effect *b*_2_ a moderately low value has been selected: *ω*_10_ = 0.6. Values for the other parameters were again the same as in Scenario 1 (cf. Table 4). More importantly, if in this situation at a given time only one input tuple occurs (either *s*_1_, *c*_1_ or *s*_2_, *c*_2_) then each action will execute according to the same behaviour as in the first scenario. The simulation of this scenario is shown in Fig. 6. The following execution trace is expected from the agent in this case:

- External stimuli *s*_1_, *s*_2_ and contexts *c*_1_, *c*_2_ will occur and trigger preparation of actions *a*_1_ and *a*_2_ separately.
- Based on the preparation state for *a*_1_ and *a*_2,_ the sensory representations of predicted effect *b*_1_ of *a*_1_ and *b*_2_ of *a*_2_ are also generated.
- Nevertheless, the agent will show strong effects on option *a*_1_ and the rate of activation for preparation of *a*_2_ will quickly slow down and disappear subsequently, due to suppression by the preparation for the other option.
- Based on this positive predicted effect and the other states for *a*_1_, a prior ownership state for action *a*_1_ is generated.
- Prior ownership for action *a*_1_ is followed by the prior awareness, generated just before the action execution.
- The agent will develop neither prior ownership nor awareness for option *a*_2_.
- Prior ownership and prior awareness states for action *a*_1_ lead to actual execution of action *a*_1_.
- The execution of *a*_1_ affects *b*_1_ in a positive manner and, via sensing, the sensory representation of *b*_1_ and the feeling of *b*_1_.
- At the same time the sensory representation of *b*_1_ is suppressed due to the prior self ownership state of it.
- Based on the generated states, after the execution of action *a*_*1*_ the agent develops a retrospective ownership state for action *a*_1_.
- Retrospective ownership of action *a*_1_ is followed by retrospective awareness of action *a*_1_.
- Finally, the agent communicates this ownership and awareness related with option *a*_1_.

The behaviour captured in the Fig. 6 is in line with the expected trace. Both action preparations [i.e. PA(*a*_*1*_) and PA(*a*_*2*_)] are activated at time point 2 and, more importantly, with the same rate of activation strength until time point 15 (this highlights the non-biased effects in early action formation). Nevertheless, after time point 15, it is clear that option *a*_1_ has maintained somewhat the same rate of activation for action preparation, while for option *a*_2_ the preparation activation strength has started to decrease, due to suppression by the preparation for option *a*_1_, via the suppressive link with strength *ω*_17_. In parallel to action preparation, the sensory representations and feelings for each option are also activated, but due to the assigned slightly lower value for weight link *ω*_10_ on option 2 the sensory representations and feelings for predicted effect of option 2 are not maintained, in addition to the effect through the suppressive link *ω*_17_ On the preparation state for *a*_2_. Each option for action preparation independently suppresses its complements’, proportional to the current strength of each preparation (as in [51] for lateral inhibition). As *ω*_10_ for option 2 is slightly weaker, this contributes to the relatively a low value of PA(*a*_2_) in comparison with PA(*a*_1_). Therefore, through these unconscious mechanisms, the activation level of preparation state PA(*a*_2_) becomes lower. Due to this bias in the action formation process, none of the other states related to the option 2 are activated. In contrast, for option 1 all the remaining states are activated in the same order as in the first scenario. More importantly, not having strong dips for sensory representation and feeling states as in the second scenario, this further highlights the conscious influence for action formation when compared to the aspects highlighted for the second scenario. The agent has developed both prior and retrospective awareness states with acceptable strength and finally has communicated the ownership and awareness specific to option 1.

As presented in the third section, this model includes some suppressive external links for the purpose of the scenario; for this scenario, once the action related to option 1 was executed it has suppressed all of the inputs, even those related to option 2 (i.e. *s*_2_, *c*_2_) and therefore all the states values become zero at the end of the simulation.

### Scenario 6: cognitive control when multiple action options compete: an extension of the fifth scenario

In the fifth scenario once the action *a*_1_ related to option 1 was executed, it has suppressed (or stopped) the input stimuli related with both the options (i.e. *s*_1_, *c*_1_, *s*_2_ and *c*_2_). In the current scenario, the same identical setup was used but execution of action *a*_1_ does not stop the input stimuli related with the second option. As the action option 2 has been suppressed by action option 1, once the effects of action option 1 have been realised, and due to that its triggers have disappeared, execution of action option 2 can get a second chance in the action formation process. The following execution trace will be expected from the agent in this case:

- External stimuli *s*_1_, *s*_2_ and contexts *c*_1_, *c*_2_ will occur in parallel and trigger preparation of actions *a*_1_ and *a*_2_.
- Based on the preparation states for *a*_1_ and *a*_2,_ the sensory representations of predicted effect *b*_1_ of *a*_1_ and *b*_2_ of *a*_2_ are generated.
- Nevertheless, the agent will show stronger effects on option *a*_1_ and the rate of activation for *a*_2_ will quickly slow down and disappear subsequently.
- Based on the positive predicted effect and the other states for *a*_1_ a prior ownership state for action *a*_1_ is generated.
- Prior ownership for action *a*_1_ is followed by prior awareness; this is generated just before the action execution.
- The agent develops neither prior ownership nor awareness for option *a*_2_.
- Prior ownership and prior awareness states for action *a*_1_ lead to actual execution of action *a*_1_.
- The execution of *a*_1_ affects *b*_1_ in a positive manner and, via sensing the sensory representation of *b*_1_ and the feeling of *b*_1_.
- At the same time, the sensory representation of *b*_1_ is suppressed due to the prior self ownership state of it.
- Based on the generated states, after the execution of action *a*_1_, the agent develops a retrospective ownership state for action *a*_1_.
- Retrospective ownership for action *a*_1_ is followed by retrospective awareness of action *a*_1_.
- Finally the agent communicates this ownership and awareness related with option *a*_1_.
- With the execution of action *a*_1_, the agent suppresses the inputs *s*_1_ and *c*_1_.
- Due to the absence of inputs *s*_1_ and *c*_1_, preparation for action *a*_1_ is not triggered anymore.
- The still existing inputs *s*_2_ and *c*_2_ still trigger preparation of action *a*_2_. This is not suppressed by preparation of action *a*_1_, since this is not activated anymore.
- Based on the preparation state for *a*_2_ the sensory representation of predicted effect *b*_2_ of *a*_2_ is generated.
- Based on this positive predicted effect and the other states a prior ownership state for action *a*_2_ is generated.
- Prior ownership for action *a*_2_ is followed by prior awareness for action *a*_2_, which is generated just before the execution of action *a*_2_.
- Prior ownership and prior awareness states for action *a*_2_ lead to actual execution of action *a*_2_.
- The execution of *a*_2_ affects *b*_2_ in a positive manner and, via sensing the sensory representation of *b*_2_ and the feeling of *b*_2_.
- At the same time, the sensory representation of *b*_2_ is suppressed due to the prior self ownership state.
- Based on the generated states, after the execution of action *a*_2_ the agent develops a retrospective ownership state for action *a*_2_.
- Retrospective ownership of action *a*_2_ is followed by retrospective awareness of action *a*_2_.
- Finally the agent communicates this new ownership and awareness too.

**Fig. 7:**
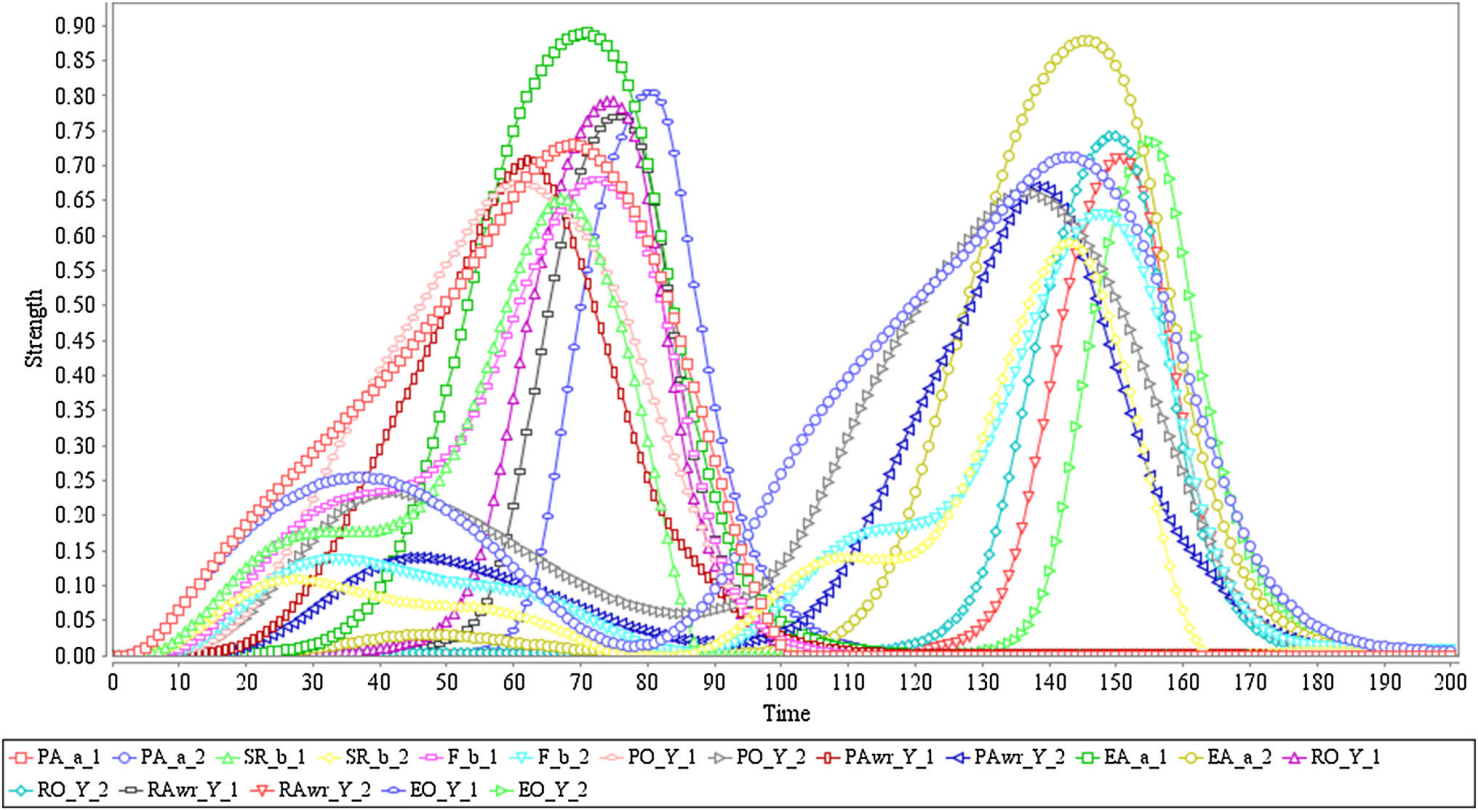
Scenario 6: Effects of cognitive controlling when multiple action options compete but after the first action execution. In here ‘Y_1’ represents ‘*a*_1_,* b*_1_,* c*_1_,* s*_1_’

The simulation of this scenario is shown in Fig. 7. In this figure, from time point 0 to (roughly) 80 the behaviour is exactly the same as in the fifth scenario, but around time point 80 due to the suppressive effect on inputs *s*_1_ and *c*_1_ the agent shows the effects of losing the suppression of action option *a*_2_. Therefore, the agent has again strengthens the preparation of action *a*_2_ and subsequently the sensory representation of predicted effect *b*_2_ of *a*_2_, and the feeling of *b*_2_. Prior ownership co-occurs with the above states (mainly due to its pre obtained activation strength). Followed by the prior ownership state, the prior awareness state develops as expected. As a result of the prior ownership and awareness states, the agent initiates the actual execution of action *a*_2_, which propagates its effects through the external body loop. This shows a possibility to get action *a*_2_ executed, in contrast to Scenario 6 above where due to the action competition it is suppressed. The peak value obtained for the action execution is 0.88 (the same for option *a*_1_ is 0.89); this clearly shows that both options have the same power in getting executed. The execution of action *a*_2_ (via the body loop) has further effects too. Due to the action execution, the agent develops the retrospective ownership state for action *a*_2_, which is followed by a retrospective awareness state. Finally, the agent communicates ownership and awareness about the just performed action. This simulation shows the ability of action selection through competition mainly from an unconscious perspective. Having a slightly different weight value for *ω*_10_ [from PAwr(*a*_*i*_ *, b*_*i*_ *, c*_*k*_ *, s*_*k*_) to PA(*a*_*i*_)] on action option 2, the same process can be simulated to demonstrate the effects of conscious cognitive control as a top–down effect. Furthermore, it is also possible to combine both of these effects in a simulation.

### Scenario 7: mismatch between the predicted and actual effects of an action

In most of the previous scenarios, the predicted effect always has positively affected action execution. Nevertheless, it may not always be the case and as pointed out in Sect. 1 (with the example of learning how to ride a bicycle) there may be a difference between what is predicted and what actually occurs. In this scenario, this phenomena will be simulated. For this simulation, a single input is considered but which triggers preparation of an action *a*_1_ for which two options for an effect are considered: the first is *b*_1_ which is the predicted effect of *a*_1_, whereas the second *b*_2_ is not predicted. The second option indicates what actually will occur after execution of *a*_1_ (see Table 4). In Table 4, the weight changes for this case have been highlighted for each effect option separately. For the effect option 1 weight *ω*_3_ [i.e. from EA(*a*_*1*_) to WS(*b*_*1*_)] was set ‘0’ as the effect of actual execution is not *b*_1_ and the same weight for effect option 2 was set ‘0.5’ to facilitate the different non-predicted effect of the action (the same explanation for *ω*_4_). The weight from WS(*b*_2_) to SS(*b*_2_) (i.e. *ω*_7_ for option 2) was set 0.5 to facilitate the effects of actual sensing (this value can be further increased but 0.5 was selected merely to highlight an average effect in the sensing). Also according to the formation of this scenario at the beginning, the agent will only predict the effect *b*_1_ of action *a*_1_, but not *b*_2_. Therefore, the weight *ω*_10_ [i.e. PA(*a*_*1*_) to SR(*b*_*2*_)] was set ‘0’ (the same explanation applies for weight *ω*_12_). The weight *ω*_30_ from EA(*a*_1_) to RO(*a*_1_, *b*_1_, *c*_1_, *s*_1_) was set ‘0’ as there will not be any retrospective ownership for action *a*_1_. Furthermore, the weight *ω*_32_ for effect option 2 [i.e. from PO(*a*_1_, *b*_2_, *c*_1_, *s*_1_) to RO(*a*_1_, *b*_2_, *c*_*1*_, *s*_1_)] was set ‘0’ as there is no prior ownership on action option *a*_2_. Additionally, the link from EA(*a*_1_) to RAwr(*a*_1_, *b*_1_, *c*_1_, *s*_1_) (i.e. *ω*_34_) was also set ‘0’. Also as there is no prior awareness related with effect option *b*_2_, weight *ω*_37_ [i.e. from PAwr(*a*_1_, *b*_2_, *c*_1_, *s*_1_) to RAwr(*a*_1_, *b*_2_, *c*_1_, *s*_1_)] was also set ‘0’. The following execution trace is expected from the agent:

- External stimulus *s*_1_ and context *c*_1_ occur and trigger preparation of action *a*_1_.
- Based on the preparation state for *a*_1_, the sensory representation of a (positive) predicted effect *b*_1_ of *a*_1_ is generated.
- Based on this positive predicted effect and the other states, a prior ownership state for action *a*_1_ is generated.
- Prior ownership for action *a*_1_ is followed by prior awareness, which is generated just before the action execution.
- Prior ownership and prior awareness states for action *a*_1_ lead to actual execution of action *a*_*1*_.
- The execution of *a*_1_ does actually not affect *b*_1_ but a different effect *b*_2_. Therefore, through sensing the agent will develop a sensory representation of *b*_2_ and the feeling of *b*_2_.
- Based on the generated states, after the execution of action *a*_1_ the agent may develop a low retrospective ownership state for action *a*_1_ with effect *b*_2_ due to the conflict between predicted effect and sensed actual effect.
- Retrospective ownership of action *a*_1_ with effect *b*_2_ is followed by retrospective awareness of action *a*_1_ with effect *b*_2_.
- Finally the agent may not properly communicate this ownership and awareness (depend on the context).

**Fig. 8:**
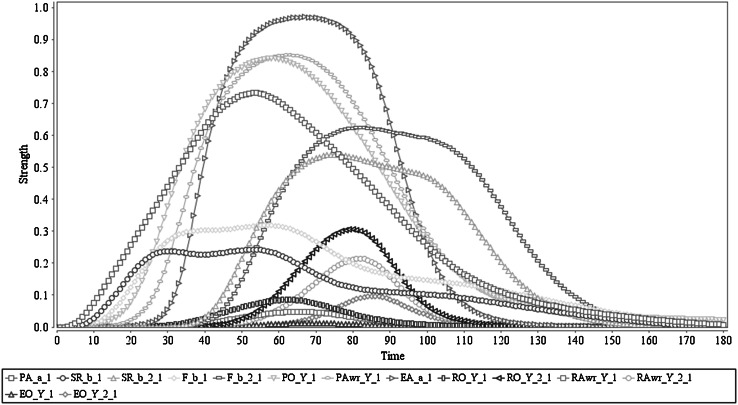
Scenario 7: Mismatch between the predicted and actual effects of an action. In here ‘Y_1’ represents ‘*a*_1_,* b*_1_,* c*_1_,* s*_1_’ whereas ‘Y_2’ represents ‘*a*_2_,* b*_2_,* c*_2_,* s*_2_’

The simulation of this scenario is shown in Fig. 8. As expected the agent triggers preparation of action *a*_1_ at time point 3 (with the peak value of 0.73). Based on that the sensory representation of predicted effect *b*_1_ of *a*_1_ is generated (through the as-if body loop with peak value 0.23), followed by the feeling of *b*_1_ (through the as-if body loop with the peak value 0.30). Next these states contribute to generate a prior ownership (for *a*_1_ with effect *b*_1_) which starts to trigger at the time point 5 with peak value 0.84. After activating the prior ownership, prior awareness develops, mainly upon the formation process of effect prediction *b*_1_ for *a*_1_. The prior awareness starts to pop up around time point 12 and obtains peak value 0.85. As a result of the prior awareness and ownership states, the agent initiates the actual execution of action *a*_1_ which propagates its actual effects through the (external) body loop. The action execution starts at time point 13 and its maximum strength is 0.97. In Fig. 8, it is clearly shown that the execution of action *a*_1_ does not affect *b*_1_ via sensing. Instead it has a different actual effect *b*_2_; through sensing it affects the sensory representation and the feeling of *b*_2_. Therefore, the sensory representation *b*_1_ of *a*_1_ does not behave as expected by adding the sensed actual effect to the predicted effect and the same effect does not propagate to the feeling of *b*_1_ (cf. [20, 47, 48]). Two activations: that do occur are of the sensory representation of *b*_2_ and feeling for *b*_2_. They emerge just at that point in time as they were not there at the beginning. These new states provide satisfactory activation levels but contribute for relatively low retrospective ownership and awareness states (mainly due to the nonexistence of influences of prior ownership and awareness states, respectively). Subsequently due to these poor retrospective ownership and awareness states, agent does not properly communicate the action and its effect. Poor communication was noted experimentally in [62] through a card game together with a covert exchange to facilitate the conflict between predicted and actual outcome. Therefore, also from experimental findings this poor communication can be justified. Furthermore, in simulation scenario 1 when there is no conflict between predicted and occurring effect, prior awareness on sensed effect has initiated at time point 35, whereas in this simulation it occurs around time point 50. Therefore, the temporal gap between the action and its perceived sensory outcome is less when the awareness is pre-existing but it is more when the (prior) awareness is not involved, which is observable from these simulation results; this is referred as intentional binding [15].

This behaviour has shown the effects when there is a conflict between what predicted versus actual. In future work, this will be especially useful when learning should incorporate with the model in a dynamical form. Having this conflict between sufficiently large prior awareness and a very low retrospective awareness can be further explored with more neuroscientific evidences. This is useful for adaptive behaviours especially for situation awareness-driven applications.

### Scenario 8: cognitive impairment for shifting to a positive feelings

In real life, agents will experience both positive and negative situations. Nevertheless, if an agent always sticks to negative thoughts while suppressing the possible positive thoughts, in the long run this will lead to a depression. In this scenario, the agent is preparing for two action options where one leads to a positive feeling F(*b*_1_), while the other leads to a negative feeling F(*b*_2_). According to the bias to negative feelings, the agent tends to select the negative action though both options share exactly the same weight values for all weights on each option, except for *ω*_29_ which is for prior awareness (which introduces a conscious intervention on action execution). For the weight *ω*_29_ on the first (positive) action option 0.2 was used and for the second (negative) option 0.8. Except this change all the other parameter values are identical and through this only the impact from biased conscious awareness towards a particular action is modelled. If the agent is executing the action which is associated to negative feeling (though at the same time a possible positive action also exists) this will affect the agent’s mood (the mood of the agent has not been in the scope of this model but will be a future work). Having a negative mood all the time together with this biased awareness towards such negative actions will take the agent to a cognitive disorder state called depression. A healthy agent will have the ability to shift in a relatively short time period again to positive actions by correcting the biased influence. Therefore, this model can be used to simulate a depression situation also, but with further improvements in the model. For the biased cognitive impairment on negative actions through awareness, the following trace is expected:

- The external stimulus *s*_1_ and context *c*_1_ will occur and trigger preparation of actions *a*_1_ and *a*_2_.
- Based on the preparation state for *a*_1,_ the sensory representation of predicted effect *b*_1_ of *a*_1_ is generated and in the same way for *a*_2,_ the sensory representation of predicted effect *b*_2_ of *a*_2_ is generated.
- It is assumed to be that both effects positively contribute to the action formation process while the effect *b*_1_ has a positive associated feeling, and the effect *b*_2_ has a negative associated feeling.
- Based on this positive predicted effect and the other states, a prior ownership state for action *a*_1_ and *a*_2_ is independently generated.
- Prior ownership for action *a*_1_ is followed by the prior awareness of *a*_1_ and the prior ownership for action *a*_2_ is followed by the prior awareness of *a*_2_.
- Nevertheless, the prior awareness of *a*_2_ will dominate the action formation process due to the biased competition.
- Due to this the states related to action *a*_1_ will loose their activation, while the sates related to action *a*_2_ will continue as the selected options.
- Prior ownership and prior awareness states for action *a*_2_ lead to actual execution of action *a*_2_.
- The execution of *a*_2_ affects *b*_2_ in a positive manner through the sensing.
- The agent develops sensory representation of *b*_2_ and the feeling of *b*_2_ in line with what is predicted.
- Based on the generated states, the agent develops a retrospective ownership state for action *a*_2_ with effect *b*_2_.
- Retrospective ownership of action *a*_2_ with sensed effect *b*_2_ is followed by retrospective awareness of action *a*_2_ with effect *b*_2_.
- Finally the agent communicates this ownership and awareness.

**Fig. 9:**
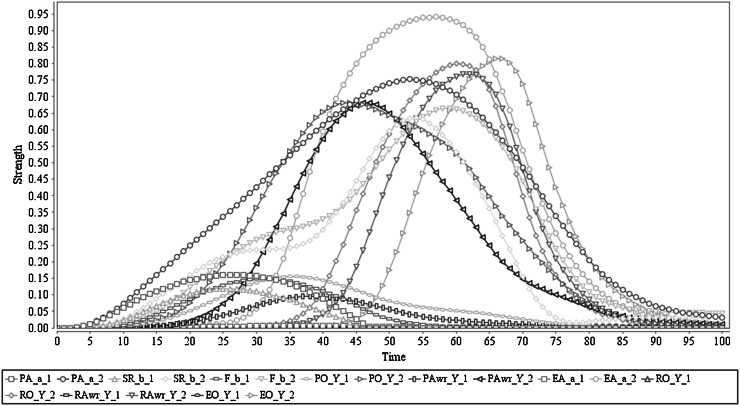
Scenario 8: Cognitive impairment for shifting to a positive feeling. In here ‘Y_1’ represents ‘*a*_1_,* b*_1_,* c*_1_,* s*_1_’ whereas ‘Y_2’ represents ‘*a*_2_,* b*_2_,* c*_2_,* s*_2_’

The simulation of this scenario is shown in Fig. 9; it is in line with the expected trace. The agent has initiated two action preparations once the input stimuli are received. Both PA(*a*_1_) and PA(*a*_2_) are activated at time point 3 and almost with the same activation speed. Parallel to this the agent also initiates the sensory representation and feelings for effects *b*_1_ and *b*_2_ for actions *a*_1_ and *a*_2_, respectively. Furthermore, the rates of activation for these four states are also almost the same at the very beginning (until prior awareness states pop up). Followed by these states, prior ownership states are also activated for both options and then subsequently the prior awareness states for each option. Roughly at time point 10, the prior awareness states start to emerge and from that very moment the states related with the option 1 show decline effects. For example, the action preparation states initially have same speed but after the time point 10 state PA(*a*_1_) is losing the speed, whereas PA(*a*_2_) maintains the same momentum and reaches maximum value 0.75. Similar to this SR(*b*_1_) reaches peak value 0.11 through the as-if body loop (i.e. through the predictive process), whereas SR(*b*_2_) reaches peak value 0.23 through the predictive process and 0.63 through the body loop. Furthermore, F(*b*_1_) reaches peak value 0.14 through the as-if body loop, whereas F(*b*_2_) reaches peak value 0.30 through the predictive process and 0.66 through the body loop. Moreover, PO(*a*_1_, *b*_1_, *c*_1_, *s*_1_) reaches peak value 0.15, whereas PO(*a*_2_, *b*_2_, *c*_2_, *s*_2_) reaches peak value 0.68. Also PAwr(*a*_1_, *b*_1_, *c*_1_, *s*_1_) reaches only a 0.09 value but PAwr(*a*_2_, *b*_2_, *c*_2_, *s*_2_) reaches 0.68.

These results clearly show the impact from conscious intervention for the action formation process (all the other unconscious related suppressive parameter values are identical for both options and therefore no bias was introduced by unconscious processes). Therefore, due to this bias the negatively associated action option *a*_2_ has executed with peak value 0.92. Furthermore, it is shown that the execution of action *a*_2_ (via the body loop) also affects in positive manner via sensing, the sensory representation of *b*_2_ and the feeling of *b*_2_. Due to these, the agent develops RO(*a*_2_, *b*_2_, *c*_2_, *s*_2_) at time point 30 with peak value 0.79 and RAwr(*a*_2_, *b*_2_, *c*_2_, *s*_2_) at the time point 34 with peak value 0.76. The agent does not develop any retrospective effects associated to action option *a*_1_. Finally, the agent has strongly communicated the ownership and awareness on action *a*_2_.

## Discussion and future work

Computational modelling is considered an important pillar for the development of cognitive science and related disciplines [63–65]. Moreover, the developments in brain imaging and recording techniques also strongly contribute to more and more focused phenomena explored in the cognitive, behavioural, affective and physiological research areas. Nevertheless, there is room for a more generic, compound process to explain a wide range of cognitive functionalities by aggregating many of these local but highly influential information from the computational perspective [66]. For example, Ron Sun in [66] has stated that “*integrative computational cognitive modelling may serve in the future as an antidote to the increasing specialization of scientific research*” [66], p. 14. On the other hand, there are many implications of these hypotheses (or evidences) and it would be beneficial if there was a mechanism that can be used to scrutinize these ideas or hypotheses using this as a workbench in much more abstract and global level (cf. [64]). Additionally, the human brain and its phenomena are immeasurably complex systems/processes that involve many uncountable factors that make experiments not always coherent with reality. Nevertheless, having computational models enables to uplift the progress of understanding these processes in a broader level as a multidisciplinary approach (cf. [63]).

From the Artificial Intelligence perspective, more and more complex systems related problems are addressed that include human cognitive aspects. For example, situations related to air traffic management, stock market analysis, business processes, human awareness of energy usage, cognitive impairments and various medical disorders, how a certain form of therapy can have its effect on a patient, etc. In many such situations, it is not practical to create real experimental setups to analyse the emergence of problems. Nevertheless, the importance and significance of such analysis is essential from a safety, performance and health perspective. Multi-agent-based simulation approaches have been noted as the potential solution for this [67] by considering agent-based simulations. Such simulations can capture emergent phenomena, providing a natural description of a system, and its flexibility: essentials for such complex system research. Even though agent-based simulations are a promising technology for this, there are many problems associated with it in terms of more realistic models for such agents to behave or perform. Often non nature inspired, simple heuristically determined rule-driven agent simulations are used for this, although reality is more complicated and far away from those simplifications. It needs more realistic representations and analysis in much closer to the natural situation (cf. [67]). Therefore, in agent systems more realistic computational models that make use of the latest neuro-cognitive findings can be used to simulate agent behaviour in a more realistic manner. Especially when it comes to the systems that include human cognition factors in dynamic systems, nature inspired cognitive computational models have more power to provide realistic results. Therefore, computational cognitive modelling as a multidisciplinary approach has many benefits for both cognitive science and artificial intelligence.

This paper presented a computational cognitive model for action awareness focusing on action preparation and performance by considering its cognitive effects and affects from both prior and retrospective perspective relative to the action execution. It is a fundamental research question what is the human cognition behind the action selection and how this is related to conscious and unconscious elements. Mainly for two hypotheses or claims, attention has been obtained by the community, as presented in Sects. 1 and 2. However, it is not yet clear what is the exact process behind this human cognition. The first hypothesis (by Benjamin Libet and others) claimed that humans may prepare for and perform actions without being conscious of these preparation and execution processes, and the awareness of motor intention of this action is not causing the behaviour, but comes after the action preparation and relatively just before the action execution time [4–8]. The second hypothesis (by Haggard and co-workers) claims that awareness of an action is a dynamic combination of both prior awareness and retrospective awareness through predictive motor control and inferential sense-making relative to the action execution, respectively [20, 21]. Furthermore, the intentional binding effect [15] shows the impact of awareness on action selection and this contributes to the second hypothesis to bring out the influence of awareness on action selection. Although these two hypothesises seem to contradict each other from the semantic point of view, by other research (cf. [5]) from a pragmatic point of view it seems hard to generally reject any of these claims on the basis of the available empirical evidences. This paper utilizes both ideas into a compound process (together with other supportive processes) and scrutinizes the behaviour through related scenarios. This work was not conducted from scratch, but adopts parts of the model presented in [23], mainly for the mechanisms of action ownership and other unconscious states/processes (e.g. action preparation, sensory representation, effect prediction process, mirroring, etc.). Having that previous model which was validated through simulations mainly for unconscious action formation, in this paper its scope is further extended to incorporate the conscious aspects related to action selection. The main research questions for this work are

1. How does the internal prediction process shape or contribute to the (prior) awareness of the action?
2. How does the inferential sense-making shape or contribute to the (retrospective) awareness of the action execution?
3. How does the awareness contribute to action execution?
4. What is the relation and interplay between conscious and unconscious action formation through action ownership and relevant awareness states?

Each research question is explored from the cognitive science perspective and analysed through the modelling perspective (with simulations) to isolate a working definition. For the internal prediction process, the hypothesis of Damasio’s as-if body loop is used as a basis, as previously presented in [23]. In the current model, it is further extended by embedding an unconscious process referred as the lateral inhibition (see [49]) to strengthen the competition among action options through the as-if body loop. With that new addition the unconscious action prediction process is coupled with the prior awareness state (as a higher order cognitive state) to facilitate the conscious aspects as proposed by Haggard et al.: predictive processes are also playing a role in action awareness (e.g. see [20, 21]). Also given empirical evidence to support the idea that awareness of motor intention for an action comes after the action preparation and relatively just before the action execution time (cf. [4–8]), in this model it is ensured that the awareness states are always higher order cognitive states which are not getting affected by most of the low level cognitive states. Predictive processes anticipate effects of each action option and lead to a competition to get selected a GO signal. The basic decision-making is assumed to be based on a feeling-related valuations associated with the effects of each action option. These internally predicted feelings work as a bottom–up feedback to develop a coherent conscious experience of action selection, mainly on action options that have strong predictive effects; therefore, actions with poor predictive effects do not get any conscious attention. Therefore, prior awareness state is only affected by the feeling and prior ownership states. The simulation results have confirmed that when there is a prior awareness it always appears just before the action execution.

The same approach is applied to the second research question, also to isolate a working definition. Once the feedback sensory information on the effects of the actual action execution is available and is integrated with the predicted effects as suggested in [20, 47, 48] to evaluate the binding of what is predicted and the actual effect. Through this sense-making process, the agent will experience the retrospective effects as presented in previous work [23], mainly for acknowledging authorship of an action, reflection on one’s own functioning, personal learning and development. This was further extended with a new abstract cognitive state name called retrospective awareness which is responsible for more conscious interpretation of retrospective effects of the action execution. Having empirical evidences for the contribution of inferential processes on action awareness from Haggard and co-workers’ experiments (cf. [20, 21]), this extension can be justified from the cognitive neuroscience perspective. Furthermore, through simulations on relevant scenarios this extension was validated. Especially the seventh scenario clearly shows that when there is a mismatch between what is predicted and what actually occurs, the two steps sigmoid behaviour on the sensory representation and the feeling states is not observed, whereas a poor retrospective awareness on the effect was predicted, as highlighted in [20]. When considering the first scenario and the seventh, this may even contribute for the observation of intentional binding (cf. [15]) mainly through the feeling state: increasing the same feeling through inferential information and forming a completely new feeling through the inferential process. In the seventh simulation, the agent does not aggregate the prior awareness to the retrospective awareness and therefore the agent will not be consciously able to communicate the conflict or mismatch. This also shows the subjective time effect found in the intentional binding: when there is no conflict between predicted and occurring effect, the prior awareness on sensed effect has initiated earlier than when a conflict occurs. Therefore, the feeling of the temporal gap between the action and its perceived sensory outcome can be explained through the retrospective awareness [15]. To have these results in this model, the retrospective awareness states were mainly affected by four states: action execution of *a*_*i*_, feeling of effect *b*_*i*_, prior awareness for action *a*_*i*_ with *b*_*i*_, *c*_*k*_, and *s*_*k*_ and prior ownership for action *a*_*i*_ with *b*_*i*_, *c*_*k*_ and *s*_*k*_.

The third research question shows many thoughts within the cognitive neuroscience community. It suggests that awareness is not a cause for an action execution but it seems like an after-effect of a set of unconscious cognitive processes leading to the action [1, 4–8]. The other idea shows that an additional influence through awareness may inject some bias or effect on decision-making especially with the findings of intentional binding [15, 21, 37, 43]. There are empirical evidences to support the second claim (at least for some inputs), emphasising why it is hard to accept the first claim due to its non moderate statistical strength on empirical evidences: in general only ~60 % accuracy in experimental data (see [5]). Therefore, this model includes both features: awareness is not required for action execution and awareness may play a role in action execution, depending on the specific scenario. In other words, the model handles both the purely unconscious action formation and the hybrid form of action formation with both conscious and unconscious elements. Furthermore, through the simulation results also this was validated. Especially the first and second scenarios provide information to show that the agent is able to execute an action with and without awareness. The eighth scenario presents a situation in which biased awareness may transform a healthy person into a cognitively impaired position. By continuing the cognition behind the eighth scenario for a longer period of time, this can even explain the effects of a patient suffering from a depression (this will be considered as a future work). Therefore, with different settings and scenarios, the model is capable to demonstrate the contribution of awareness from zero to high. Therefore, the model facilitates a good spectrum to represent the effects of awareness on action selection. There are situations, for example like flight or fight situations, which mostly show the unconscious action formation side of the spectrum that includes very quick and strong action executions (cf. [68–70]). According to the second scenario, it is clear that when agent is purely performing in an unconscious mode, the strength of action execution is relatively weak. Therefore, it may be possible to add improvements to this model and its settings, especially on the unconscious perspective including the emotion related effects. Further information on the interplay between bottom–up and top–down processes seems to be useful for such further improvements of this model (see [68–71]).

The fourth research question is realised as an aggregation of the other three questions. The interplay between conscious and unconscious action formation is mainly realised through having effects from feeling and ownership states on the awareness states (unconscious to conscious), and effects from prior awareness states to the action preparation and action execution states (conscious to unconscious). The first links (bottom–up) play a role to pass low level information to develop awareness of what is going on in a high level form, whereas the second type of links (top–down) contributes to inject some bias or excitement to intentionally drive the action formation. When there is poor activation of the bottom–up links, agent is unable to develop prior awareness and therefore, as presented in the second scenario, the agent can perform the action selection in purely unconscious form by the feeling of ownership. When the agent is having sufficient activations in bottom–up links, awareness develops and shows how that leads to an action execution, as in the first scenario. Also particularly in the eighth scenario, the role of top–down links demonstrates the power of an intentional focus on action selection. The third simulation highlights why agents are not always performing a task even in unconscious mode. This shows that even in unconscious form it is necessary to have a sufficiently large satisfactory predicted effect to have a GO signal. Therefore, this shows the mechanism of vetoing in unconscious form. The cognitive control is a useful process to explain the interplay between conscious and unconscious action formation. The fifth and sixth simulations present the role of cognitive control. In the fifth simulation, it shows how a particular option is getting suppressed by the other option through cognitive control just by having a value of 0.2 difference for the links between action preparation to sensory representation for two options. The sixth simulation shows that once the dominant option of the fifth simulation completed, the suppressed action option emerges due to not having the effects of cognitive control and, more importantly, with strong activations for each state. Through this, it is clear from the simulation perspective that the model can have different configurations to facilitate different behaviours both from the unconscious and the combination of conscious and unconscious forms. Also as mentioned in the other three research questions, the cognitive neuroscience basis behind this model was inspired by the experiments and evidences found in the literature.

Having interesting simulation results for many scenarios, still there is more to improve on this work. It was possible to isolate a generic parameter value set that worked for 8 simulations. A generic parameter value set provides more confidence from the model validation perspective and its practical usage in future complex applications. Nevertheless, it is not a trivial task to find such generic parameter value set. The approach used to identify this parameter value set was explained in Sect. 4.1, but it is not a fully automated process. Due to the complexity of the human brain and limitations in measuring techniques of human cognition, there are no detailed numerical empirical data to use in this process, but only some characteristics of behaviours are available in more fuzzy form. Due to this issue, it is not possible to directly use parameter estimation techniques available for dynamic systems. Therefore, it is useful to explore the parameter estimation techniques particular to the characteristics of these types of work. Furthermore, from the cognitive neuroscience perspective, this model has many more areas to explore both in conscious and unconscious levels. In the third scenario, it shows the vetoing process in unconscious form, but the same process with awareness which is referred as intentional inhibition is a future work for this model. Human awareness has its specializations, for example, emotional awareness, situation awareness. Working processes behind these concepts are more complicated and need further research to incorporate those into this model.

Finally, this model may be useful in many applications. Especially for agent-based simulations on complex systems that need action selection related to cognitive aspects. Also, this model can be used as a basis for subsequent work in developing ambient agent systems able to monitor, analyse and support persons trying to develop a healthy lifestyle. If such systems have such a model of the underlying human processes, they can use this to have a deeper understanding of the human.
